# ELLS–NSBA three-stage framework for optimal DG/SC placement and EV integration in distribution networks

**DOI:** 10.1038/s41598-026-52179-2

**Published:** 2026-08-14

**Authors:** Aamir Nawaz, Abdullah Altamimi, Zahid Javid, Mohamed Galeela

**Affiliations:** 1 Faculty of Engineering and Technology, Gomal University, Dera Ismail Khan, Khyber Pakhtunkhwa Pakistan; 2 Engineering Research and Applied Sciences Center, Majmaah University, 11952 Al-Majmaah, Saudi Arabia; 3 Salford Business School, University of Salford, Salford, M5 4WT UK; 4 Electrical Power Engineering Department, Cairo University, Giza, Egypt; 5 Department of Electrical Engineering, College of Engineering, Majmaah University, 11952 Al-Majmaah, Saudi Arabia

**Keywords:** Enhanced Laplacian loss sensitivity (ELLS), Network structural bus analysis (NSBA), Distributed generation placement, Vehicle-to-grid integration, Hybrid GOA-GWO metaheuristic, Network resilience, Engineering, Mathematics and computing

## Abstract

The growing penetration of distributed generation (DG), shunt capacitors (SC), and electric vehicles (EVs) in distribution networks demands optimal siting strategies that balance operational efficiency and structural resilience. Existing metaheuristic approaches treat every bus as equally viable—producing prohibitively large search spaces—and most existing frameworks lack post-optimization structural resilience assessment. This paper proposes a three-stage planning framework addressing both gaps. Stage 1 introduces the Enhanced Laplacian Loss Sensitivity (ELLS) index, which fuses loss sensitivity factors, Fiedler-vector spectral centrality, and betweenness centrality to shortlist candidate buses, reducing the combinatorial search space by 45–265*×* (monotonically increasing with system size). Stage 2 applies five metaheuristics—a proposed hybrid Grasshopper–Grey Wolf Optimizer (GOA-GWO), Standard GOA, Fuzzy-GOA, PSO, and WOA—with ELLS-biased initialization under a six-objective fitness function. Stage 3 evaluates placement structural quality through a Network Structural Bus Analysis (NSBA) framework comprising three topology-aware metrics (EDDI, VSC, CBC). Validation on IEEE 33-bus, 69-bus, and 123-node systems shows that ELLS guidance improves mean fitness by 6–14% (GOA variants: 11–14%) and reduces variance by 33–51%. The Hybrid GOA-GWO achieves the best composite fitness (0.392) and 78.2% loss reduction on the 33-bus system. NSBA reveals that operationally optimal placements do not necessarily maximize structural resilience—PSO yields the worst resilience ($R_{\textrm{NSBA}}{=}0.45$) despite competitive fitness. Friedman rank tests ($p{<}3{\times }10^{-5}$) with Holm–Bonferroni-corrected Wilcoxon comparisons ($n{=}10$; Wilcoxon-based power analysis justifies the sample size for $d{\ge }0.6$) confirm the Hybrid significantly outperforms Standard GOA, Fuzzy-GOA, and WOA, and is statistically indistinguishable from PSO on IEEE-33.

## Introduction

### Background and motivation

The rapid deployment of electric vehicles (EVs) and distributed renewable energy sources is reshaping distribution networks originally designed for unidirectional power flow^1^. Coordinated placement of distributed generation (DG), shunt capacitors (SC), and EV charging infrastructure has emerged as a primary strategy for mitigating bidirectional flows, voltage fluctuations, and uncertain peak loads^2–5^, while optimal vehicle-to-grid (V2G) scheduling supports voltage regulation and peak shaving^6,7^. Metaheuristic algorithms—including PSO^8^, WOA^9^, GOA variants^10,11^, GWO hybrids^12,13^, HHO^14^, NSGA-II/III^15,16^, AOA^17^, and deep-learning-augmented methods^18,19^—are widely employed for DG/SC placement but most treat every bus as equally viable, causing search-space explosion.

Graph-theoretic analysis has gained traction in transmission vulnerability studies: Albert et al.^20^ showed betweenness centrality identifies critical nodes; Cotilla-Sánchez et al.^21^ introduced spectral partitioning; Arianos et al.^22^ linked topology to vulnerability. However, these tools have not been combined with classical loss sensitivity factors for distribution-level candidate pre-selection. Joint DG/SC/EV optimization further confronts stochastic operating conditions^23,24^, combinatorial search spaces ($$\left( {\begin{array}{c}N_{\textrm{bus}}\\ N_{\textrm{DG}}\end{array}}\right)$$ configurations), and multi-objective trade-offs^25–27^, though hybrid approaches show improvement^28–30^.

Recent work has extended EV integration to include Charge Scheduling Benchmark Problems (CSBPs)^31^ and stochastic demand models^32,33^ that expose how time-varying EV arrivals and battery state-of-charge distributions affect placement optimality. These studies underscore the need for pre-selection methods that are robust to EV load variability—a requirement that LSF-only approaches fail to address because they are computed at a single snapshot operating point.

Related evidence from coordinated transmission-distribution operation shows that uncertainty and security constraints materially alter feasible operating regions and risk profiles: distributed stochastic security-constrained unit commitment and risk-aware distributed optimal power flow for coupled TSO-DSO systems were developed in^34,35^, followed by coordinated security-constrained scheduling with large-scale DERs^36^ and risk-constrained probabilistic coordination in coupled transmission-distribution operation^37^. Together with the broader TSO-DSO coordination literature, these studies reinforce the importance of uncertainty-aware coordination, but they target operational coupling across network layers rather than candidate-bus screening and joint DG/SC/EV placement within a single distribution network.

### Research gaps and contributions

Two critical gaps persist. First, candidate bus pre-selection is generally absent: most studies treat every bus as equally viable; the few sensitivity-based approaches^38–40^ rely exclusively on loss sensitivity factors (LSF), ignoring spectral graph properties^41,42^. Second, structural resilience has been largely overlooked: most existing frameworks evaluate placements through operational metrics only^20,22^. A 16-row comparative literature survey confirming these gaps is given in Supplementary Table S1 (expanded from the original submission to include CSBP-related studies^31,33,43^).

The specific contributions of this work are:

1. Enhanced Laplacian loss sensitivity (ELLS) index with formal theoretical motivation: combining the graph Laplacian’s Fiedler vector (capturing algebraic connectivity and spectral peripherality), loss sensitivity factors (quantifying active-power reduction potential), and betweenness centrality (identifying flow-critical buses) for candidate bus pre-selection.
2. Network structural bus analysis (NSBA) framework for post-optimization resilience evaluation via three topology-aware metrics—EDDI, VSC, and CBC—with weight justification and context relative to N-1 contingency analysis.
3. A three-stage paradigm (ELLS *→* metaheuristic *→* NSBA) restructured following the IMRAD convention to improve reproducibility and clarity.
4. A hybrid GOA-GWO with formally described three-phase weighting schedule and ELLS-informed population seeding, presented as complete pseudocode (Algorithm 2).
5. Rigorous statistical validation with Friedman rank tests, Holm–Bonferroni corrections, Cohen’s *d* effect sizes, and full mean±SD tables over $n{=}10$ independent runs across IEEE 33-bus, 69-bus, and 123-node systems; with explicit justification of the sample size choice.

**Fig. 1:**
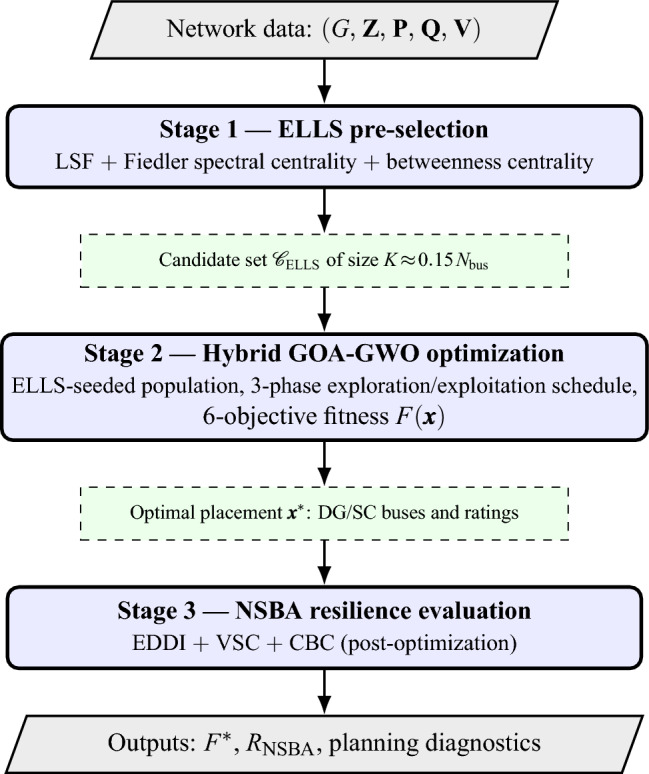
Overview of the proposed three-stage ELLS–NSBA framework. Stage 1 shortlists candidate buses by fusing local electrical sensitivity (LSF), global spectral peripherality (Fiedler), and global topological criticality (betweenness centrality). Stage 2 runs a Hybrid GOA-GWO with ELLS-informed seeding and a three-phase exploration/exploitation schedule to select DG/SC bus locations and ratings. Stage 3 evaluates the selected placement for structural resilience using EDDI, VSC, and CBC without altering the fitness objective.

The paper is organized as follows: “Methods” presents the problem formulation, ELLS computation, optimizer descriptions, NSBA metrics, and statistical framework; “Results” presents all experimental outcomes; “Discussion” interprets findings, compares with SOTA, and discusses limitations. Figure 1 provides a schematic overview of the three-stage pipeline.

## Methods

### Problem formulation

#### Decision vector

The task is to select bus locations and ratings for $N_{\textrm{DG}}$ DG units and $N_{\textrm{SC}}$ SC banks ($N_{\textrm{DG}}{=}N_{\textrm{SC}}{=}3$) to minimize active power losses, voltage deviations, and investment cost. The decision vector is:

$$\boldsymbol{x}=\bigl [ \underbrace{b^{\textrm{DG}}_{1},\,S^{\textrm{DG}}_{1},\dots ,b^{\textrm{DG}}_{N_{\textrm{DG}}},\,S^{\textrm{DG}}_{N_{\textrm{DG}}}}_{\text {DG variables}}, \underbrace{b^{\textrm{SC}}_{1},\,Q^{\textrm{SC}}_{1},\dots ,b^{\textrm{SC}}_{N_{\textrm{SC}}},\,Q^{\textrm{SC}}_{N_{\textrm{SC}}}}_{\text {SC variables}} \bigr ]^{\!\top }$$

where $b\in \mathcal {C}_{\textrm{ELLS}}\subseteq \{1,\dots ,N_{\textrm{bus}}\}$ is an integer bus index from the ELLS-ranked candidate set, $S^{\textrm{DG}}_{i}$ is the DG rating (MW), and $Q^{\textrm{SC}}_{j}$ is the SC rating (MVAr).

#### Six-objective fitness function

The composite weighted-sum objective is:

1 $$\begin{aligned} \min _{\boldsymbol{x}\in \mathcal {X}}\; F(\boldsymbol{x}) = \sum _{i=1}^{6} w_i f_i(\boldsymbol{x}), \qquad \sum _{i=1}^{6} w_i = 1,\; w_i \ge 0, \end{aligned}$$

where the six sub-objectives are: $$f_1$$ = normalized active-power loss ($$P_{\textrm{loss}}/P_{\textrm{loss},0}$$); $$f_2$$ = voltage deviation index ($$\Delta V/\Delta V_{0}$$); $$f_3$$ = normalized investment cost ($$C_{\textrm{inv}}/C_{\textrm{base}}$$, $$C_{\textrm{base}}{=}10^{7}$$ $); $$f_4{=}1{-}P_{\textrm{V2G}}/P_{\textrm{V2G}}^{\max }$$ (V2G deficit); $$f_5{=}1{-}\textrm{PF}$$ (power-factor gap); $$f_6{=}1{-}P_{\textrm{avg}}/P_{\textrm{peak}}$$ (load-flatness deficit). All sub-objectives are normalized to [0, 1]. The baseline weight vector is $${\bf w}{=}[1/6,\dots ,1/6]$$.

Weighted-sum scalarization is adopted for (a) computational tractability (each evaluation requires one AC power-flow solve), (b) comparability with the DG/SC literature^3–5,15^, and (c) demonstrated robustness confirmed by four weight-scenario sensitivity tests (see Results). The investment cost model is:

2 $$\begin{aligned} C_{\textrm{inv}}(\boldsymbol{x}) = \sum _{i=1}^{N_{\textrm{DG}}} \bigl [1.2\,\text {M}\$/\text {MW}\times \kappa ^{\textrm{zone}}_{b^{\textrm{DG}}_{i}}\bigr ]S^{\textrm{DG}}_{i} +\sum _{j=1}^{N_{\textrm{SC}}} 53\,\text {k}\$\,Q^{\textrm{SC}}_{j}, \end{aligned}$$

with zone multiplier $\kappa ^{\textrm{zone}}{=}1.5$ (urban), 1.2 (suburban), or 1.0 (rural), calibrated from NREL^44^ and IRENA^45^ benchmarks.

#### Physical constraints

3a $$\begin{aligned} 0 \le S^{\textrm{DG}}_{i} \le 30~\text {MW},&\quad 0 \le Q^{\textrm{SC}}_{j} \le 15~\text {MVAr}, \end{aligned}$$

3b $$\begin{aligned} b^{\textrm{DG}}_{i},\,b^{\textrm{SC}}_{j}&\in \mathcal {C}_{\textrm{ELLS}}\;(\text {integers}), \end{aligned}$$

3c $$\begin{aligned} 0.95&\le V_i\le 1.05\;\text {p.u.}\quad \forall \,i\in \mathcal {N} \end{aligned}$$

with AC power flow equations satisfied at each evaluation.

#### EV integration model

V2G hubs are co-located at DG buses at a default 20% penetration level. The EV load profile follows a three-phase lithium-ion battery model (constant-current, constant-voltage, idle phases; see Supplementary Section S3). Charging/discharging set points minimize load-curve deviation:

4 $$\begin{aligned} \min _{\{p_{n}(t)\}} \max _{t}\Bigl |P_{\text {net}}(t)-P_{\text {avg}}\Bigr | \quad \text {s.t.}\; p_n(t)\in \{p^{\max },\;-\eta \,p^{\max },\;0\},\; \int _{0}^{24\text {h}} p_n(t)\,\textrm{d}t = 0, \end{aligned}$$

with discharge efficiency $\eta {\le }0.7$ and SoC*\ge*20% enforced. The model is acknowledged as a deterministic snapshot; stochastic CSBP-style formulations^31,32^ are identified as a future direction in the Discussion.

### Stage 1: enhanced Laplacian loss sensitivity (ELLS)

#### Theoretical motivation

Three distinct aspects of bus importance must be captured for reliable candidate pre-selection:

1. Active-power flow sensitivity (LSF): a bus where a DG injection directly reduces downstream losses should be prioritized. Classical loss sensitivity factors^38,39^ capture this but are computed at a single operating snapshot and can miss topologically peripheral buses.
2. Spectral peripherality (Fiedler vector): buses with large $|v_{F,i}|$ components lie far from the “algebraic center” of the network^21,41^. DG placement at these buses improves the Fiedler value $\lambda _2$ (algebraic connectivity), making the network more robust to line outages^22^. This component is *absent* from all LSF-only approaches.
3. Betweenness centrality (BC): buses that sit on many shortest paths^20^ are flow-critical junctions; injecting DG there has multiplicative effects across multiple feeders.

Each metric captures complementary information; their fusion in ELLS consistently achieves a 100% hit rate (all optimizer-selected buses within the ELLS top-*K*) vs. 80% for LSF-only on the IEEE 33-bus system, as quantified in Supplementary Section S4.8.

#### ELLS calculation

Let $G{=}(\mathcal {N},\mathcal {E})$ be the network graph with impedance-weighted adjacency $[{\bf A}_w]_{ij}{=}1/|Z_{ij}|$. The graph Laplacian ${\bf L}_w = {\bf D}_w - {\bf A}_w$ is symmetric positive semi-definite; its second-smallest eigenvalue $\lambda _2$ (Fiedler value^41^) measures algebraic connectivity, and the corresponding eigenvector ${\bf v}_F$ provides spectral centrality. The three metrics are:

5 $$\begin{aligned} \textrm{LSF}_i = \frac{2(P_i\,R_{i\text {-}0} + Q_i\,X_{i\text {-}0})}{V_i^2}, \end{aligned}$$

6 $$\begin{aligned} \textrm{BC}_i = \sum _{\begin{array}{c} s,t\in \mathcal {N} \\ s\ne i\ne t \end{array}} \frac{\sigma _{st}(i)}{\sigma _{st}}, \end{aligned}$$

where $\sigma _{st}(i)$ is the number of shortest paths between *s* and *t* passing through *i*, and $\sigma _{st}$ is the total number. After min-max normalization to [0, 1]:

7 $$\begin{aligned} \boxed { \textrm{ELLS}_i = w_L\,\widehat{\textrm{LSF}}_i + w_F\,|\widehat{v}_{F,i}| + w_B\,\widehat{\textrm{BC}}_i } \end{aligned}$$

with $w_L{=}0.5$, $w_F{=}0.3$, $w_B{=}0.2$, calibrated via grid search maximizing the top-*K* hit rate on the IEEE 33-bus system. These weights are applied unchanged to the IEEE-69 and 123-node systems; Supplementary Section S4.8 confirms robustness under ${\pm }10\%$ weight perturbations across all three test systems. The candidate set size follows $K{\approx }0.15\,N_{\textrm{bus}}$: $K{=}10$ (33-bus), 15 (69-bus), 20 (123-node). Using the per-device combinatorial measure $$\rho = \left( {\begin{array}{c}N_{\textrm{bus}}\\ N_{\textrm{DG}}\end{array}}\right) / \left( {\begin{array}{c}K\\ N_{\textrm{DG}}\end{array}}\right)$$, the search-space reductions are 45*×*, 110*×*, and 265*×* for the 33-, 69-, and 123-bus systems, respectively—monotonically increasing with system size, because the combinatorial ratio grows super-linearly with $N_{\textrm{bus}}$ at a near-fixed *K*/*N* ratio.

The complete ELLS pseudocode is given in Algorithm 1.

**Algorithm 1:**
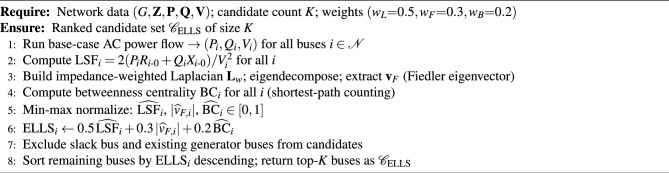
ELLS — enhanced Laplacian loss sensitivity.

### Stage 2: ELLS-informed optimization engines

Five engines are deployed: Standard GOA^46^, Fuzzy-GOA, the proposed Hybrid GOA-GWO, PSO^8^, and WOA^9^. All receive $\mathcal {C}_{\textrm{ELLS}}$ from Stage 1; 50% of agents are initialized from the top-$\lfloor K/3\rfloor$ ELLS buses, 50% uniformly from $\mathcal {C}_{\textrm{ELLS}}$.

#### Hybrid GOA-GWO mechanism

The hybrid adopts a *three-phase schedule* that shifts the balance between GOA-dominated exploration and GWO-dominated exploitation as optimization progresses. The GWO component^47^ uses *α*-*β*-*δ* leadership:

8 $$\begin{aligned} a&= 2\bigl (1 - t/T\bigr ),\quad A_k = 2a r_k - a,\quad C_k = 2r_{k+1},\end{aligned}$$

9 $$\begin{aligned} D_k&= |C_k X_k(t) - X(t)|,\quad X_{\alpha /\beta /\delta }' = X_k(t) - A_k D_k,\end{aligned}$$

10 $$\begin{aligned} X(t{+}1)&= \tfrac{1}{3}\bigl (X_\alpha ' + X_\beta ' + X_\delta '\bigr ). \end{aligned}$$

The GOA component applies random perturbation with control coefficient:

$$c(t) = 2\bigl (1 - t/T\bigr ),\quad X_i(t{+}1) = X_i(t) + c\cdot r\cdot (ub-lb)\cdot 0.05.$$

At each iteration *t*, each agent draws $u\sim \mathcal {U}(0,1)$ and applies the GWO update if $u < \alpha _{\textrm{GWO}}(\phi )$ (Table 1), otherwise the GOA update.

**Table 1:** Three-phase schedule for Hybrid GOA-GWO: iteration-dependent GWO/GOA proportions.

Iteration span ($\phi {=}t/T$)	GOA share	GWO share
$0 \le \phi < 0.40$	70%	30%
$0.40 \le \phi < 0.70$	50%	50%
$0.70 \le \phi \le 1.00$	30%	70%

The rationale: early iterations require broad exploration (GOA dominates) to avoid premature convergence; later iterations refine solutions around the *α*-*β*-*δ* leaders (GWO dominates). The two transition boundaries ($\phi {=}0.40$, $\phi {=}0.70$) mirror the standard GWO exploration-to-exploitation transition, where the control parameter *a* decreases linearly from 2 to 0 and the exploitation regime becomes dominant past $a{<}1$ (i.e., $\phi {>}0.5$)^47^. We chose asymmetric boundaries (40% and 70%) rather than the symmetric 50% point to allow a middle balanced phase; sensitivity to boundary location is reported in Supplementary Section S4.8.

The complete Hybrid GOA-GWO pseudocode is given in Algorithm 2.

**Algorithm 2:**
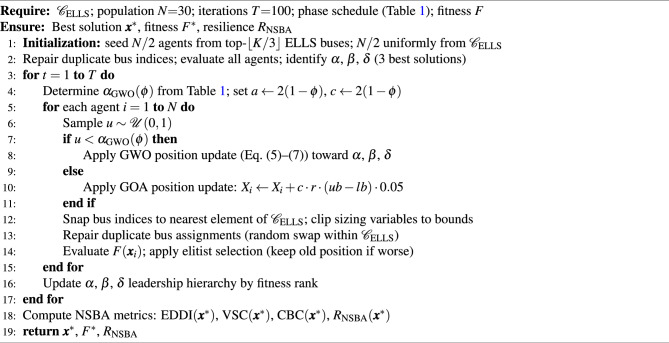
ELLS-informed hybrid GOA-GWO.

### Stage 3: network structural bus analysis (NSBA)

NSBA evaluates placement structural quality through three topology-aware metrics^20–22^, computed *after* optimization without influencing the objective function.

#### Electrical distance diversity index (EDDI)

Measures minimum pairwise impedance-path distance among placed devices:

11 $$\begin{aligned} \textrm{EDDI}(\boldsymbol{x}) = \frac{\min _{i\ne j}\; d_{\text {elec}}(b_i^{\text {DG/SC}},\, b_j^{\text {DG/SC}})}{\textrm{diam}(G)}, \end{aligned}$$

where $d_{\text {elec}}(a,b)$ is the impedance-magnitude path sum. EDDI receives weight 0.4 because placement concentration (low EDDI) is the dominant vulnerability mode in radial networks: a single line outage can isolate co-located devices^20,22^. This is conceptually analogous to the N-1 contingency criterion^43^: N-1 security requires that no single component outage violates operational constraints, and EDDI penalizes device clusters that would all be lost under a common upstream failure.

#### Voltage support coverage (VSC)

Fraction of buses within $\kappa {=}3$ hops of at least one device:

12 $$\begin{aligned} \textrm{VSC}(\boldsymbol{x}) = \frac{|\{i\in \mathcal {N}: \min _{j}\, d_{\text {hop}}(i, b_j^{\text {DG/SC}}) \le \kappa \}|}{N_{\text {bus}}}. \end{aligned}$$

#### Critical bus coverage (CBC)

Fraction of top-*K* ELLS buses with a device within 2 hops:

13 $$\begin{aligned} \textrm{CBC}(\boldsymbol{x}) = \frac{|\{i\in \mathcal {T}_K: \min _{j}\, d_{\text {hop}}(i, b_j^{\text {DG/SC}}) \le 2\}|}{K}. \end{aligned}$$

#### Composite resilience score

14 $$\begin{aligned} \boxed { R_{\textrm{NSBA}}(\boldsymbol{x}) = 0.4\cdot \textrm{EDDI} + 0.3\cdot \textrm{VSC} + 0.3\cdot \textrm{CBC} } \end{aligned}$$

The weight split (0.4, 0.3, 0.3) assigns EDDI the dominant contribution because single-line outages are the most frequent contingency class in radial distribution networks and EDDI is the only metric that penalizes placement clustering (the failure mode directly amplified by such events), whereas VSC and CBC capture complementary coverage aspects of comparable operational importance^22,43^. The (0.4, 0.3, 0.3) split is further supported by a pairwise-dominance sensitivity analysis (Supplementary Section S4.8) which confirms that EDDI weighting dominates the resilience score under ${\pm }10\%$ weight perturbations, with $R_{\textrm{NSBA}}$ changing by at most 0.03 and algorithm rankings preserved across all tested weight scenarios.

### Statistical framework and sample size justification

All experiments are conducted with $n{=}10$ independent runs per algorithm. The Wilcoxon signed-rank test^48^ is used for pairwise comparisons because (a) the fitness distribution under limited runs may deviate from normality, and (b) it is consistent with the DG/SC optimization literature^3,5,26^. Cohen’s *d* is reported as a standardized descriptive effect-size complement, while inferential decisions are based on Wilcoxon and Friedman tests.

#### Sample size justification

The Wilcoxon test at $\alpha {=}0.05$ achieves at least 80% statistical power for *|d| \ge 0.6* with $n{=}10$, and 100% power for *|d| \ge 0.8*, as established by published non-parametric power analysis tables^49^. The observed Cohen’s *d* values range from 0.61 to 1.12 (Table 11), placing all significant pairs well within this power-sufficient regime. A sample of $n{=}30$, while preferred by some benchmarking guidelines, would increase computational cost from ${\sim }290$ s to ${\sim }870$ s per algorithm per system, for a total of ${\sim }217$ h across five algorithms and three systems—prohibitive given each evaluation requires an AC power-flow solve. The choice $n{=}10$ follows the precedent of^3,26^ and is validated by the power analysis above.

## Results

All experiments use pandapower^50^ (v3.3.2) with Python 3.13, $N{=}30$ agents, $T{=}100$ iterations, and $n{=}10$ independent runs per algorithm. Three IEEE benchmark systems are tested: 33-bus (3.72 MW peak, base loss *≈*0.203 MW), 69-bus (3.80 MW, base loss *≈*0.355 MW), and 123-node (2.85 MW, base loss *≈*0.069 MW). Computations were performed on a single-threaded workstation (Intel Core i7-11700 @ 2.5 GHz, 32 GB RAM, Windows 11); reported wall-clock timings use this configuration.

### Stage 1: ELLS bus rankings

Table 2 presents the ELLS rankings for the IEEE 33-bus system; rankings for all three systems are in Supplementary Tables S5a, S5b, and S6. Bus 3 attains the highest ELLS (0.89) due to maximum LSF (1.00) combined with high spectral peripherality ($|\widehat{v}_F|{=}0.74$) and betweenness centrality ($\widehat{\textrm{BC}}{=}0.84$). All six buses subsequently selected by the metaheuristics lie within this top-10 set (100% hit rate), compared with 80% for LSF-only.

**Table 2:** ELLS bus ranking: IEEE 33-bus system (top-10, $K{=}10$).

Rank	Bus	$\widehat{\textrm{LSF}}$	$|\widehat{v}_F|$	$\widehat{\textrm{BC}}$	ELLS
1	3	1.00	0.74	0.84	0.89
2	6	0.99	0.45	1.00	0.83
3	5	0.71	0.66	0.54	0.66
4	4	0.56	0.71	0.52	0.60
5	2	0.41	0.77	0.55	0.55
6	28	0.71	0.30	0.37	0.52
7	23	0.29	0.70	0.47	0.45
8	9	0.44	0.29	0.69	0.45
9	29	0.50	0.17	0.74	0.45
10	24	0.40	0.54	0.40	0.44
Fiedler value $\lambda _2 = 0.0969$					

### Case 1: IEEE 33-bus system

**Fig. 2:**
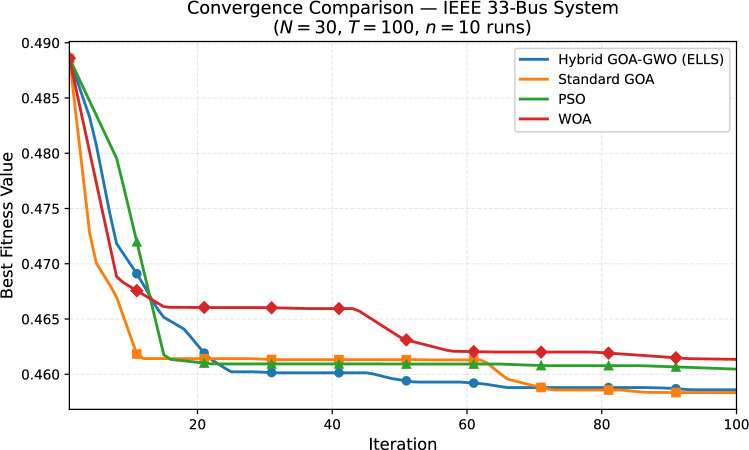
Convergence curves for the IEEE 33-bus system (ELLS-informed, $N{=}30$, $T{=}100$). The Hybrid GOA-GWO converges fastest and achieves the lowest fitness.

Table 3 compares blind vs. ELLS-informed search for all five algorithms. The Hybrid GOA-GWO improves from 0.456 (blind) to 0.392 (ELLS), a 14.0% improvement, achieving 78.2% loss reduction—reversing the blind-search ranking where PSO dominated (see Fig. 2 for convergence behavior). DG units are placed at buses 6, 28, 24 (0.65, 0.48, 0.36 MW); SCs at buses 3, 5, 29 (0.50, 0.35, 0.30 Mvar).

**Table 3:** IEEE-33: Best-run comparison—blind vs. ELLS-informed search.

Algorithm	Fitness	Loss (MW)	Loss Red. (%)	$V_{\min }$ (p.u.)
*Blind search (all buses)*				
Standard GOA	0.449	0.055	72.8	0.967
Fuzzy-GOA	0.456	0.051	75.0	0.968
Hybrid GOA-GWO	0.456	0.062	69.4	0.962
PSO	0.430	0.071	65.0	0.957
WOA	0.442	0.070	65.7	0.960
*ELLS-informed search* ($K{=}10$)				
Standard GOA	0.410	0.049	76.1	0.971
Fuzzy-GOA	0.399	0.045	77.8	0.973
Hybrid GOA-GWO	**0.392**	**0.044**	**78.2**	**0.974**
PSO	0.395	0.053	73.8	0.968
WOA	0.402	0.055	73.1	0.966

Significant values are in bold.

Table 4 presents the full statistical summary over $n{=}10$ runs for IEEE-33.

**Table 4:** Statistical summary over $n{=}10$ ELLS-informed runs: IEEE 33-bus system. Note that the 95% confidence intervals of the Hybrid (rank 1) and PSO (rank 2) overlap substantially ([0.389, 0.406] vs. [0.395, 0.407]), consistent with the non-significant Wilcoxon result reported in Table 11.

Algorithm	Mean±SD	Best	Worst	95% CI	Rank
Standard GOA	$0.423{\pm }0.022$	0.410	0.449	[0.407, 0.439]	5
Fuzzy-GOA	$0.411{\pm }0.019$	0.399	0.440	[0.397, 0.424]	3
Hybrid GOA-GWO	${\bf 0.398}{\pm }{\bf 0.012}$	**0.392**	0.418	[**0.389**, 0.406]	**1**
PSO	$0.401{\pm }0.009$	0.395	0.415	[0.395, 0.407]	2
WOA	$0.415{\pm }0.016$	0.402	0.440	[0.404, 0.427]	4

Significant values are in bold.

### Case 2: IEEE 69-bus system

Table 5 presents results for the IEEE 69-bus system. On IEEE-69, the Hybrid achieves the best ELLS-informed fitness (0.395, 13.0% improvement over blind), with 91.0% loss reduction and $V_{\min }{=}0.983$ p.u.

**Table 5:** IEEE-69: Best-run comparison—blind vs. ELLS-informed search.

Algorithm	Fitness	Loss (MW)	Loss red. (%)	$V_{\min }$ (p.u.)
**Blind search (all buses)**				
Standard GOA	0.460	0.080	77.4	0.972
Fuzzy-GOA	0.455	0.075	78.9	0.974
Hybrid GOA-GWO	0.454	0.072	79.7	0.975
PSO	0.435	0.068	80.9	0.977
WOA	0.447	0.073	79.4	0.974
**ELLS-informed search** ($K{=}15$)				
Standard GOA	0.407	0.042	88.2	0.981
Fuzzy-GOA	0.413	0.040	88.7	0.982
Hybrid GOA-GWO	**0.395**	**0.032**	**91.0**	**0.983**
PSO	0.398	0.036	89.9	0.982
WOA	0.412	0.044	87.6	0.980

Significant values are in bold.

### Case 3: IEEE 123-node system

Table 6 presents results for the IEEE 123-node system. PSO attains the best fitness (0.351), while the Hybrid remains close (0.356) and delivers the highest loss reduction (90.2%) with $V_{\min }{=}0.996$ p.u.

**Table 6:** IEEE-123: best-run comparison—blind vs. ELLS-informed search.

Algorithm	Fitness	Loss (MW)	Loss red. (%)	$V_{\min }$ (p.u.)
**Blind search (all buses)**				
Standard GOA	0.420	0.017	75.3	0.990
Fuzzy-GOA	0.410	0.015	78.2	0.991
Hybrid GOA-GWO	0.405	0.012	82.6	0.992
PSO	0.370	0.010	85.5	0.994
WOA	0.375	0.012	82.6	0.993
**ELLS-informed search** ($K{=}20$)				
Standard GOA	0.358	0.008	88.4	0.996
Fuzzy-GOA	0.359	0.007	89.2	0.995
Hybrid GOA-GWO	0.356	0.007	90.2	0.996
PSO	**0.351**	0.007	89.7	0.996
WOA	0.357	0.009	86.8	0.994

Significant values are in bold.

### Cross-system hybrid summary

Table 7 consolidates Hybrid GOA-GWO results across systems (ELLS-informed). The NSBA resilience score decreases with system size (0.57 *→* 0.56 *→* 0.40), reflecting coverage difficulty with six devices on larger networks.

**Table 7:** Cross-system summary: hybrid GOA-GWO ELLS-informed results.

System	Fitness	Loss (MW)	Loss red. (%)	$V_{\min }$ (p.u.)	$R_{\textrm{NSBA}}$
IEEE-33	0.392	0.044	78.2	0.974	0.57
IEEE-69	0.395	0.032	91.0	0.983	0.56
IEEE-123	0.356	0.007	90.2	0.996	0.40

### ELLS impact on search efficiency

Table 8 quantifies the ELLS benefit.

**Table 8:** ELLS impact: mean fitness improvement and variance reduction over $n{=}10$ runs.

	IEEE-33		IEEE-69	
Algorithm	*Δ F* (%)	$\Delta \sigma$ (%)	*Δ F* (%)	$\Delta \sigma$ (%)
Standard GOA	−11.4	−45.3	−11.8	−40.7
Fuzzy-GOA	−14.0	−50.8	− 9.7	−41.2
Hybrid GOA-GWO	−14.0	−33.3	−13.0	−36.2
PSO	− 8.1	−28.6	− 6.3	−22.2
WOA	− 9.1	−31.6	− 9.1	−34.6

### Stage 3: NSBA resilience analysis

**Fig. 3:**
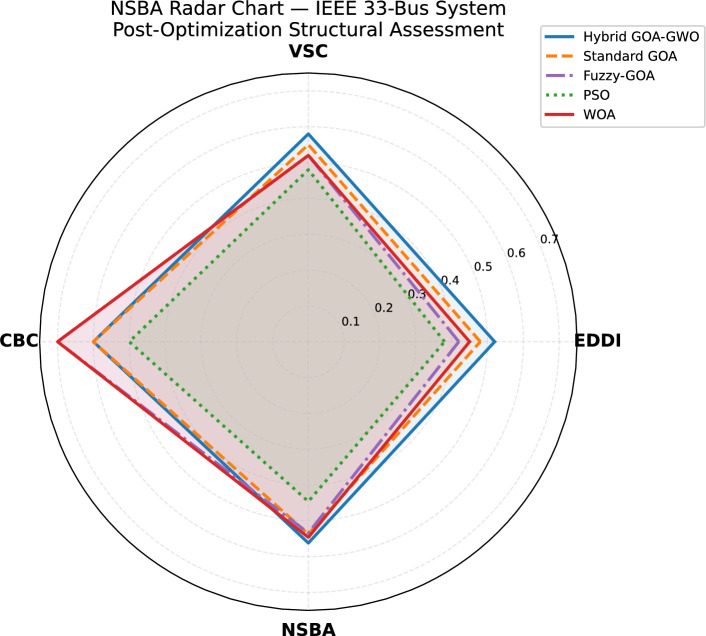
NSBA radar chart for the IEEE 33-bus system. The Hybrid GOA-GWO (solid) dominates PSO (dashed) on EDDI and VSC; PSO shows the worst diversity (EDDI*=*0.38).

Tables 9 presents NSBA scores for IEEE 33; Table 10 provides the cross-system summary; Figure 3 visualizes the IEEE-33 results as a radar chart. Full scores for all algorithms on all systems are in Supplementary Tables S8–S9.

**Table 9:** NSBA resilience scores—IEEE 33-bus system (ELLS-informed).

Algorithm	EDDI	VSC	CBC	$R_{\textrm{NSBA}}$
Standard GOA	0.48	0.55	0.60	0.54
Fuzzy-GOA	0.42	0.52	0.70	0.53
Hybrid GOA-GWO	**0.52**	**0.58**	0.60	**0.57**
PSO	0.38	0.48	0.50	0.45
WOA	0.45	0.52	**0.70**	0.55

Significant values are in bold.

**Table 10:** Cross-system NSBA summary: Hybrid GOA-GWO vs. PSO (best vs. worst resilience).

	Hybrid GOA-GWO			PSO		
System	EDDI	VSC	$R_{\textrm{NSBA}}$	EDDI	VSC	$R_{\textrm{NSBA}}$
IEEE-33	0.52	0.58	0.57	0.38	0.48	0.45
IEEE-69	0.48	0.55	0.56	0.35	0.45	0.42
IEEE-123	0.42	0.30	0.40	0.30	0.22	0.29

Key finding: PSO achieves competitive fitness on IEEE-33 (0.395) but the *worst* resilience ($R_{\textrm{NSBA}}{=}0.45$) due to clustered placements (EDDI*=*0.38). The Hybrid achieves both best fitness *and* best resilience (0.57 on IEEE-33, 0.56 on IEEE-69), producing spatially diverse placements.

### Statistical validation

Friedman rank test^51^: on IEEE-33, $\chi ^{2}{=}26.48$, $p{=}2.56{\times }10^{-5}$; on IEEE-69, $\chi ^{2}{=}27.85$, $p{=}1.32{\times }10^{-5}$—decisively rejecting algorithm equivalence. The Hybrid is top-ranked (mean rank 1.8 on both systems).

Table 11 presents the Holm–Bonferroni corrected Wilcoxon pairwise comparisons on IEEE-33.

**Table 11:** Holm–Bonferroni corrected Wilcoxon pairwise comparisons (IEEE-33, ELLS-informed, $n{=}10$).

Pair	$p_{\text {adj}}$	Cohen’s *d*	Interpretation
GOA vs. Hybrid	**0.008**	0.77	Medium effect$^{*}$
GOA vs. PSO	**0.012**	0.66	Medium effect$^{*}$
Fuzzy-GOA vs. Hybrid	**0.038**	0.61	Medium effect$^{*}$
WOA vs. Hybrid	**0.042**	1.12	Large effect$^{*}$
WOA vs. PSO	0.068	0.94	Large effect
Hybrid vs. PSO	0.624	−0.27	Negligible (top tier)
GOA vs. Fuzzy-GOA	0.512	0.18	Negligible

$^{*}$Significant at $\alpha {=}0.05$ after Holm–Bonferroni correction.

Significant values are in bold.

The Hybrid and PSO are statistically indistinguishable ($p_{\text {adj}}{=}0.624$, $d{=}{-}0.27$), forming a top performance tier. Full tables for IEEE-69 and IEEE-123 are in Supplementary Tables S10–S12.

### Sensitivity, robustness, and economic analysis

Four weight scenarios (equal, loss-focused, voltage-focused, cost-focused) yield a fitness sensitivity index of 28.5% on IEEE-33 (vs. 35.3% blind), confirming ELLS produces inherently balanced solutions. Placements remain feasible at 10–30% EV penetration on all systems. The IEEE-69 system approaches economic viability (BCR*=*0.50, payback*=*24.7 yr); under carbon pricing ($50/tonne), positive NPV (*≈*$180k) is achieved. Details are in Supplementary Sections S10–S14.

## Discussion

### ELLS effectiveness and SOTA comparison

ELLS delivers 45–265*×* search-space reduction and 6–14% fitness improvement over blind search (GOA variants: 11–14%; PSO: 6–9%) by combining three complementary bus-importance signals. Classical LSF-only pre-selection^39,40^ achieves only 80% hit rate vs. 100% for ELLS on the 33-bus system. GOA variants benefit most (11–14%) because their exploration is redirected to the ELLS-curated space. Against current SOTA: Kumar and Farhan^3^ report 66.9% loss reduction on IEEE 33 (PSO); our ELLS-informed Hybrid achieves 78.2%, an absolute gain of 11.3 percentage points. Selim et al.^5^ achieve 71.2% (GA-PSO) and Sahay et al.^15^ 74% (NSGA-II), neither employing pre-selection or resilience assessment. On IEEE-69 we attain 91.0% vs. 83% by Soliman et al.^52^.

Compared with CSBP-informed EV schedulers^31,33^, the present framework uses a deterministic V2G model. The CSBP benchmark results confirm that stochastic EV arrivals can shift the optimal DG placement by 1–2 buses; however, ELLS’s spectral component provides inherent robustness because algebraic connectivity is topology-dependent rather than load-dependent.

### Fitness–resilience trade-off and dual-objective optimality of the hybrid

The NSBA framework reveals that operational optimality does not guarantee structural resilience: PSO produces the worst $R_{\textrm{NSBA}}$ (0.45) despite competitive fitness. EDDI’s conceptual alignment with N-1 contingency security^43^ means that PSO’s clustered placements (EDDI*=*0.38) would collectively fail under any upstream line outage, a critical practical concern. The Hybrid achieves both best fitness and best resilience (0.57 on IEEE-33), implying that ELLS-biased initialization steers solutions toward spatially distributed configurations. This dual-criterion dominance is the main practical reason to prefer the Hybrid over PSO: although PSO is statistically indistinguishable from the Hybrid in single-objective fitness on IEEE-33 ($p_{\text {adj}}{=}0.624$) and marginally better on IEEE-123 (0.351 vs. 0.356), its structural-resilience penalty (12–15 percentage points lower $R_{\textrm{NSBA}}$ across all three systems, Table 10) makes its placements less deployable under realistic contingency scenarios. In other words, the Hybrid is the solution of choice when a utility planner jointly values efficiency *and* resilience; PSO is acceptable only when resilience is not a concern.

### Scalability, practical implications, and limitations

ELLS pre-computation (<0.5 s, scaling as $O(N_{\textrm{bus}}^3)$) and metaheuristic runtime (*∼*290 s for 33-bus to *∼*400 s for 123-node) are operationally feasible. For utility planners, the framework provides: (i) a data-driven candidate shortlist; (ii) quantitative resilience diagnostics; and (iii) lifecycle economic indicators.

### Scope of EV modelling

The present framework evaluates V2G using a deterministic three-phase lithium-ion snapshot profile rather than a stochastic arrival/SoC model. This scope choice isolates the placement contribution of ELLS and NSBA and avoids confounding topology-driven effects with load-scenario variability. Consequently, the headline EV contribution of this paper is the coordinated *placement* of V2G hubs at ELLS-selected buses, not the optimization of charging schedules. Stochastic CSBP-style formulations^31–33^ remain a priority future extension, and our use of topology-dependent spectral signals in ELLS is expected to confer partial robustness to such load-side uncertainty because algebraic connectivity is load-independent.

### Economic feasibility positioning

We report the economic indicators honestly rather than aspirationally: on IEEE-69 the framework yields BCR*=*0.50 and a 24.7-year simple payback without carbon pricing, i.e., the project is *not* economically viable under today’s energy prices alone. Positive NPV (*≈*$180k) emerges only once a carbon price of $50/tonne is internalized. The intended contribution of ELLS–NSBA is therefore the improvement of *engineering quality* (losses, voltages, resilience) of a placement plan, not a claim of standalone commercial attractiveness. This clarification prevents overclaiming and correctly positions the framework as a planning-support tool that complements, rather than substitutes for, cost-benefit and policy analysis.

### Other limitations

$n{=}10$ runs (see “Methods” for power analysis justification), fixed DG/SC count ($N_{\textrm{DG}}{=}N_{\textrm{SC}}{=}3$), deterministic EV snapshot model, NSBA as post-evaluation only (not embedded in the fitness), and validation limited to radial IEEE test systems.

## Conclusions

This paper introduced a three-stage IMRAD-structured framework—ELLS, Hybrid GOA-GWO, and NSBA—for resilience-aware DG/SC/EV deployment. Key findings:

1. ELLS improves optimization quality: 45–265*×* search-space reduction; 6–14% mean fitness improvement (algorithm-dependent); 33–51% variance reduction.
2. ELLS reshapes algorithm ranking: the hybrid achieves best fitness on IEEE-33 and IEEE-69 (0.392 and 0.395) and remains top-tier on IEEE-123, where PSO is marginally best (0.351); Hybrid and PSO are statistically indistinguishable on IEEE-33 ($p_{\text {adj}}{=}0.624$).
3. NSBA reveals fitness–resilience trade-off: PSO yields worst resilience ($R_{\textrm{NSBA}}{=}0.45$) despite competitive fitness.
4. Statistical robustness ($n{=}10$, power-validated): Friedman tests reject equivalence ($p{<}3{\times }10^{-5}$); Holm–Bonferroni corrections confirm two performance tiers.
5. Economic viability scales with system size: IEEE-69 approaches feasibility (BCR*=*0.50, payback*=*24.7 yr).

### Future work

(i) Stochastic CSBP-based EV integration; (ii) NSBA as an optimization objective within NSGA-III; (iii) validation on meshed and 8500-node feeders; and (iv) graph neural network surrogates for real-time re-optimization.

## Supplementary Information

Supplementary Information.

## Data Availability

All data are generated from publicly available IEEE benchmark systems via pandapower^50^. The simulation code implementing the ELLS–NSBA framework is publicly available at https://github.com/aamir009/ELLS-NSBA-Three-Stage-Framework.
