# Supplementary material for: ELLS–NSBA three-stage framework for optimal DG/SC placement and EV integration in distribution networks
Source: Sci Rep. 2026 Aug 14;16:25456. doi: 10.1038/s41598-026-52179-2 (PMC13473527; doi:10.1038/s41598-026-52179-2)
Supplement: Supplementary file 1 [file 41598_2026_52179_MOESM1_ESM.pdf]

Supplementary Material for: ELLS–NSBA Three-Stage Framework for Optimal DG/SC Placement and EV Integration in Distribution Networks

Aamir Nawaz[1], Abdullah Altamimi[2,*], Zahid Javid[3], and Mohamed Galeela[4]

[1]Faculty of Engineering and Technology, Gomal University, Dera Ismail Khan, Khyber Pakhtunkhwa, Pakistan
[2]Department of Electrical Engineering, College of Engineering, Majmaah University, Al-Majmaah 11952, Saudi Arabia
[3]Salford Business School, University of Salford, The Crescent, Salford M5 4WT, England, UK
[4]Electrical Power Engineering Department, Cairo University, Giza, Egypt
[*]a.altmimi@mu.edu.sa

ABSTRACT

This supplement provides extended tables, detailed algorithm formulations, pseudocode, and additional analyses referenced in the main paper. Section S0 provides the nomenclature table. Section S1 presents the full literature comparison table. Section S2 details the enhanced land acquisition cost model. Section S3 describes the electric vehicle charging model. Section S4 provides individual algorithm formulations (GOA, Fuzzy-GOA, GWO, PSO, WOA), phase-based weightings, parameter tables, and complete pseudocode for ELLS (Algorithm S1) and the Hybrid GOA-GWO (Algorithm S2). Sections S5–S6 contain ELLS rankings for all three systems and IEEE 123-node optimization results. Sections S7–S10 report extended statistical validation. Sections S11–S14 present weight sensitivity, EV penetration, economic, ablation, and convergence analyses. Section S15 provides extended discussion. Section S16 contains additional figures (ELLS bar chart, NSBA radar chart, convergence curves, voltage profiles) and tables (placement details, best-fit comparisons, ELLS impact, NSBA scores, and statistical summaries) from the main paper.

S0. Nomenclature

Table 1. Table S0: Nomenclature — symbols and abbreviations used in the main paper

Symbol	Description	Symbol	Description
b_i^{DG}	Bus index for i-th DG	S_i^{DG}	Rating of i-th DG (MW)
Q_j^{SC}	Rating of j-th SC (MVAr)	P_{loss}	Active power loss (MW)
ΔV	Voltage deviation index	C_{inv}	Investment cost ($)
$\mathbf{L}_w$	Impedance-weighted graph Laplacian	λ_2	Fiedler value (algebraic connectivity)
$\mathbf{v}_F$	Fiedler eigenvector	LSF_i	Loss sensitivity factor at bus i
BC_i	Betweenness centrality at bus i	ELLS_i	Enhanced Laplacian Loss Sensitivity score
$\mathscr{C}_{\text{ELLS}}$	ELLS-ranked candidate bus set	K	Number of ELLS candidate buses
R_{NSBA}	NSBA composite resilience score	EDDI	Electrical Distance Diversity Index
VSC	Voltage Support Coverage	CBC	Critical Bus Coverage
$N_{\text{DG}}, N_{\text{SC}}$	Number of DG/SC units (= 3)	N, T	Population size, iterations
w_i	Objective weight for f_i	$\phi(t)$	Iteration progress t/T

S1. Literature Comparison

Table 2 benchmarks recent DG/SC/EV optimization studies (2022–2025) against the present work across eight critical dimensions.

No existing study simultaneously addresses all eight dimensions. Most works omit candidate bus pre-selection entirely, relying on blind search over all buses; none incorporates a structural resilience assessment. Even the sensitivity-based

Table 2. Table S1: Comparative overview of recent DG/SC/EV optimization studies vs. the proposed framework

Reference	Year	Method	Test Sys.	DG+SC	EV	Stats	Pre-sel.	Resil.
Wang et al.[1]	2024	Compet. Alg.	33	✓	✓	×	×	×
Kumar & Farhan[2]	2025	PSO	33, 69	✓	✓	×	×	×
Selim et al.[3]	2023	GA-PSO	33, 69	✓	×	×	×	×
Jayabarathi et al.[4]	2024	Plant Growth	33, 69	✓	×	×	×	×
Gurumoorthi et al.[5]	2024	GWO-GOA	33	✓	×	×	×	×
Alruqimi & Rahmat[6]	2024	DL + PSO	33	×	×	×	×	×
Sahay et al.[7]	2025	NSGA-II	33	✓	✓	×	×	×
Singh et al.[8]	2025	DE-GWO	33, 69	✓	×	×	×	×
Heidari et al.[9]	2019	HHO	33, 69	✓	×	×	×	×
Abualigah et al.[10]	2021	AOA	33	✓	×	×	×	×
Mahdavi et al.[11]	2023	NSGA-III	33, 69	✓	✓	✓	×	×
Murthy & Kumar[12]	2013	LSF + GA	33, 69	✓	×	×	✓	×
Hung et al.[13]	2014	Analytical	33, 69	×	×	×	✓	×
**This work**	**2025**	**ELLS + GOA-GWO**	**33, 69, 123**	✓	✓	✓	✓	✓

studies[12, 13] use only classical LSF without topological enrichment. The present framework is the first to combine spectral-graph-enriched candidate pre-selection (ELLS), joint DG+SC+V2G co-optimization, NSBA resilience assessment, lifecycle economic modeling, formal statistical validation, and cross-topology benchmarking.

S2. Enhanced Land Acquisition Cost Model

Total land cost at location i is:

$$C_{\text{land},i} = A_i \, P_{\text{land},i} \, M_{\text{zone},i} \, M_{\text{scar},i} + C_{\text{prep},i},$$

where A_i is the required acreage (3.5 ac/MW for PV, 5.0 ac/MW for wind, 1.5 ac/MW for fast charging), $P_{\text{land},i}$ is the base land price, $M_{\text{zone},i}$ is the zone-dependent multiplier, $M_{\text{scar},i}$ captures land scarcity premiums, and $C_{\text{prep},i}$ covers site preparation costs.

Table 3. Table S2: Zone-dependent land multipliers used in the cost model

Zone	$/acre (median)	M_{zone}
Urban core	200 k	8.4
Urban	110 k	3.7
Suburban	55 k	2.1
Rural	18 k	1.5

These zone-dependent multipliers are derived from median installed solar PV costs reported in NREL's Annual Technology Baseline[14] and IRENA's Renewable Power Generation Costs[15], which document 1.3–1.6× cost premiums for urban versus rural utility-scale installations due to land acquisition, permitting, and grid interconnection expenses.

S3. Electric Vehicle Charging–Load Model

The nodal EV load is represented by a three-phase profile mimicking lithium-ion battery behavior:

- **Constant–current phase** (0–4 h): charging power ramps linearly from 0 to $p^{\max}$ (11 kW);
- **Constant–voltage phase** (4–5 h): power decays exponentially as the battery approaches full charge; and
- **Idle phase** (t>5 h): $p(t)$=0.

For a fleet of N_{EV} vehicles connected at bus k, the aggregate real power demand is:

$$P_k^{\text{EV}}(t) = \sum_{n=1}^{N_{\text{EV}}(k)} p_n(t) \text{ [kW]},$$

injected into the AC load flow during each optimization iteration. V2G hubs are co-located at the DG buses selected by the optimizer. The EV fleet is distributed equally among the three hubs; at the default 20% penetration level, each hub serves $N_{\text{EV}}/3$ vehicles.

V2G scheduling framework. Charging/discharging set points are determined by a time-of-use optimizer:

$$\underset{\{p_n(t)\}}{\text{minimize}} \max_t \left| P_{\text{net}}(t) - P_{\text{avg}} \right| \quad \text{s.t. } p_n(t) \in \{p^{\max},\ -\eta\, p^{\max},\ 0\},\ \int_0^{24\text{h}} p_n(t)\,\mathrm{d}t = 0.$$

Optimal control law. For every EV n:

$$p_n(t) = \begin{cases} p^{\max} & t \in \mathscr{O}_{\text{charge}} \\ -\eta\, p^{\max} & t \in \mathscr{O}_{\text{discharge}} \\ 0 & \text{otherwise} \end{cases} \qquad \left(\eta \leq 0.7\right),$$

with the zero-energy constraint $\int_0^{24\text{h}} p_n(t)\,\mathrm{d}t = 0$. The scheme maximizes the load flattening index $L_{\text{flat}}{=}P_{\text{avg}}/P_{\text{peak}}$.

Optimization framework implementation. Each candidate solution (chromosome) encodes twelve decision variables:

$$\underbrace{(x_1,x_3,x_5)}_{\text{DG buses}}\ \underbrace{(x_2,x_4,x_6)}_{\text{DG sizes (MW)}}\ \underbrace{(x_7,x_9,x_{11})}_{\text{SC buses}}\ \underbrace{(x_8,x_{10},x_{12})}_{\text{SC sizes (MVAr)}},$$

with repair and clipping operators keeping them within physical bounds. A feasibility-preserving swap prevents duplicate bus indices. Bus indices are restricted to $\mathscr{C}_{\text{ELLS}}$, transforming the combinatorial structure from N_{bus}-ary to K-ary selection.

S4. Individual Algorithm Formulations

S4.1 Standard Grasshopper Optimization Algorithm (GOA)

Parameterization: Population size 30 agents; maximum iterations 100; comfort zone coefficient $c(t){=}1{-}t/T_{\max}$. Integer bus indices (restricted to $\mathscr{C}_{\text{ELLS}}$) are rounded after each position update; velocities are clipped to one tenth of the search range.

S4.2 Fuzzy Logic Enhanced GOA

A Mamdani fuzzy inference system with three inputs (iteration progress, fitness improvement, diversity) outputs $\Delta c \in [-0.2, +0.2]$. Table 4 shows the complete 3×3×3 rule base.

Table 4. Table S3: Complete fuzzy rule base for adaptive parameter control (Δc output)

Iteration	Fitness Improvement		
**Progress**	**Low**	**Medium**	**High**
**Diversity: Low**			
Early	Negative	Negative	Zero
Mid	Negative	Zero	Positive
Late	Zero	Positive	Positive
**Diversity: Medium**			
Early	Negative	Zero	Positive
Mid	Zero	Zero	Positive
Late	Positive	Positive	Positive
**Diversity: High**			
Early	Zero	Positive	Positive
Mid	Positive	Positive	Positive
Late	Positive	Positive	Positive

S4.3 GWO Component

The convergence parameter a decreases linearly from 2 to 0: $a = 2 - 2(t/T)$. Each search agent updates its position using information from the alpha (α), beta (β), and delta (δ) wolves:

$$A_i = 2a \cdot r_i - a, \quad C_i = 2 \cdot r_{i+1}, \tag{1}$$

$$D_i = |C_i \cdot X_i(t) - X(t)|, \tag{2}$$

$$X_i = X_i(t) - A_i \cdot D_i. \tag{3}$$

The final updated position is $X(t{+}1) = (X_\alpha + X_\beta + X_\delta)/3$.

S4.4 GOA Component

The position of each agent is updated as:

$$X_i(t+1) = X_i(t) + c \cdot r \cdot (ub - lb) \cdot 0.05,$$

where $c = 2 - 2(t/T)$ decreases linearly, $r \in [-1, 1]$, and ub, lb are the search bounds (restricted to $\mathscr{C}_{\text{ELLS}}$ for bus variables).

S4.5 Particle Swarm Optimization (PSO)

PSO is included as a well-established baseline[16]. Each particle i maintains a position $\mathbf{x}_i$ and velocity $\mathbf{v}_i$, updated as:

$$\mathbf{v}_i(t+1) = w\,\mathbf{v}_i(t) + c_1 r_1 \big(\mathbf{p}_i - \mathbf{x}_i(t)\big) + c_2 r_2 \big(\mathbf{g} - \mathbf{x}_i(t)\big), \tag{4}$$
$$\mathbf{x}_i(t+1) = \mathbf{x}_i(t) + \mathbf{v}_i(t+1), \tag{5}$$

where w (inertia) decreases from 0.9 to 0.4, c_1=c_2=2.0. Bus dimensions are restricted to $\mathscr{C}_{\text{ELLS}}$ with ELLS-informed initialization.

S4.6 Whale Optimization Algorithm (WOA)

WOA emulates bubble-net hunting[17] with three mechanisms: encircling prey (p<0.5, $|A|$<1), spiral update (p≥0.5), and random exploration ($|A|$≥1):

$$\mathbf{x}(t+1) = \mathbf{x}^*(t) - A\,\big|C\,\mathbf{x}^*(t) - \mathbf{x}(t)\big| \tag{6}$$
$$\mathbf{x}(t+1) = \mathbf{x}^*(t) + D'\,e^{bl}\cos(2\pi l). \tag{7}$$

As with all algorithms, bus indices are restricted to $\mathscr{C}_{\text{ELLS}}$.

S4.7 Hybrid GOA-GWO Parameters

Table 5. Table S4: Hybrid GOA-GWO parameters

**Parameter**	**Value**	**Description**
n_{agents}	30	Number of agents in the population
max_{iter}	100	Maximum number of iterations
a	$2 \to 0$	GWO convergence parameter
c	$2 \to 0$	GOA control coefficient
P_{hybrid}	0.5	Base probability of selecting GWO
Bus candidates	$\mathscr{C}_{\text{ELLS}}$ (ELLS top-K buses)	
Sizing	$\{1, 3, 5, 7, 9, 11\}$	Continuous decision variables

S4.7a Phase-Based GOA/GWO Weightings

Table 6. Table S4a: Phase-based GOA/GWO weightings for the Hybrid GOA-GWO

Iteration span	GOA share	GWO share
0–40%	70%	30%
40–70%	50%	50%
70–100%	30%	70%

The three-phase schedule gradually shifts from GOA-dominated exploration (70% GOA in early iterations) to GWO-dominated exploitation (70% GWO in late iterations), with a balanced transition phase. This schedule was determined empirically and is detailed in the main paper.

S4.8 Algorithm S1: ELLS Pseudocode

S4.9 Algorithm S2: ELLS-Informed Hybrid GOA-GWO Pseudocode

S5. ELLS Bus Rankings: IEEE 33-Bus, 69-Bus, and 123-Node Systems

IEEE 33-bus analysis. Bus 3 attains the highest ELLS (0.89) due to maximum LSF (1.00) combined with high spectral peripherality (0.74) and BC (0.84). Bus 6 ranks 2nd with maximum BC (1.00), recognizing its role as the junction to lateral

Algorithm 1 Algorithm S1: ELLS — Enhanced Laplacian Loss Sensitivity

Require: Network data $(G, \mathbf{Z}, \mathbf{P}, \mathbf{Q}, \mathbf{V})$; candidate count K; weights (w_L, w_F, w_B)
Ensure: Ranked candidate set $\mathscr{C}_{\text{ELLS}}$ of size K

Run base-case AC power flow $\rightarrow (P_i, Q_i, V_i)$
Compute LSF_i for all buses
Construct impedance-weighted $\mathbf{L}_w$; eigendecompose; extract Fiedler vector $\mathbf{v}_F$
Compute BC_i for all buses
Min-max normalize all three metrics to $[0, 1]$
Compute $\text{ELLS}_i = w_L \widehat{\text{LSF}}_i + w_F |\hat{v}_{F,i}| + w_B \widehat{\text{BC}}_i$
Sort buses by ELLS_i descending; exclude slack/generator buses
return top-K buses as $\mathscr{C}_{\text{ELLS}}$

Algorithm 2 Algorithm S2: ELLS-Informed Hybrid GOA-GWO

Require: $\mathscr{C}_{\text{ELLS}}$, population N=30, iterations T=100, bounds, phase schedule (Table 5)
Ensure: Best solution $\mathbf{x}^*$, fitness F^*, resilience R_{NSBA}

Initialise $N/2$ agents from top-$\lfloor K/3 \rfloor$ ELLS buses; $N/2$ uniformly from $\mathscr{C}_{\text{ELLS}}$
Repair duplicate bus indices; evaluate all agents; identify α, β, δ (GWO leaders)
for $t = 1$ **to** T **do**
    Set phase weights: GOA/GWO shares based on iteration progress
    Update convergence parameter $a = 2(1 - t/T)$; GOA control $c = 2(1 - t/T)$
    for each agent i **do**
        Draw $u \sim \mathscr{U}(0, 1)$; apply GWO update if $u < \alpha_{\text{GWO}}(\phi)$, else GOA update
        Map bus indices to $\mathscr{C}_{\text{ELLS}}$; clip continuous sizes within bounds; repair duplicates
        Evaluate fitness; keep previous position if fitness worsened (elitist selection)
    end for
    Update α, β, δ leadership hierarchy
end for
Compute $R_{\text{NSBA}}(\mathbf{x}_\alpha)$ via Stage 3 NSBA metrics
return $\mathbf{x}^*$, F^*, R_{NSBA}

Table 7. Table S5a: ELLS bus ranking for the IEEE 33-bus system (top-10, K=10)

**Rank**	**Bus**	$\widehat{\text{LSF}}$	$\lvert\hat{v}_F\rvert$	$\widehat{\text{BC}}$	**ELLS**
1	3	1.00	0.74	0.84	0.89
2	6	0.99	0.45	1.00	0.83
3	5	0.71	0.66	0.54	0.66
4	4	0.56	0.71	0.52	0.60
5	2	0.41	0.77	0.55	0.55
6	28	0.71	0.30	0.37	0.52
7	23	0.29	0.70	0.47	0.45
8	9	0.44	0.29	0.69	0.45
9	29	0.50	0.17	0.74	0.45
10	24	0.40	0.54	0.40	0.44

Fiedler value $\lambda_2 = 0.0969$

branches. This exemplifies how ELLS captures complementary structural importance: Bus 3 for flow sensitivity, Bus 6 for topological intermediacy, and Bus 28 for deep-feeder loss mitigation. All six buses selected by the metaheuristics lie within this top-10 set.

IEEE 69-bus analysis. The 69-bus system has a longer main feeder and more pronounced peripheral branches. Bus 61 achieves the highest ELLS score (0.80), combining maximum LSF (1.00) with high spectral peripherality (0.91). Bus 17 ranks 5th due to its high BC (0.88)—it is a critical trunk junction. The lower Fiedler value (λ_2=0.0132 vs. 0.0969 for the 33-bus) reflects weaker algebraic connectivity, making the 69-bus system more vulnerable to line outages and more sensitive to DG placement location.

IEEE 123-node analysis. The 123-node system is a 4.16 kV radial distribution feeder with deeper branching than the 69-bus

Table 8. Table S5b: ELLS bus ranking for the IEEE 69-bus system (top-10 of K=15)

Rank	Bus	$\widehat{\text{LSF}}$	$\lvert\widehat{v}_F\rvert$	$\widehat{\text{BC}}$	ELLS
1	61	1.00	0.91	0.11	0.80
2	62	0.96	0.95	0.04	0.78
3	65	0.87	0.98	0.03	0.74
4	64	0.90	0.93	0.05	0.74
5	17	0.72	0.55	0.88	0.71
6	50	0.81	0.68	0.32	0.67
7	27	0.68	0.61	0.57	0.64
8	49	0.75	0.63	0.28	0.63
9	12	0.58	0.42	0.73	0.57
10	21	0.55	0.50	0.45	0.52
Fiedler value $\lambda_2 = 0.0132$					

Table 9. Table S6: ELLS bus ranking for the IEEE 123-node system (top-10 of K=20)

Rank	Bus	$\widehat{\text{LSF}}$	$\lvert\widehat{v}_F\rvert$	$\widehat{\text{BC}}$	ELLS
1	66	0.93	0.24	1.00	0.72
2	115	0.87	0.77	0.38	0.68
3	34	0.83	0.23	0.90	0.65
4	39	0.88	0.25	0.81	0.65
5	118	0.87	0.92	0.14	0.64
6	117	0.87	0.88	0.18	0.64
7	119	0.86	0.95	0.11	0.64
8	116	0.87	0.83	0.21	0.64
9	120	0.86	0.98	0.07	0.64
10	107	0.99	0.43	0.47	0.63
Fiedler value $\lambda_2 = 0.1608$					

system. ELLS scores are tightly clustered (range 0.63–0.72 for top 10) compared to the smaller radial systems, because the deeper tree structure distributes loss sensitivity more evenly across branches. Bus 66 ranks first (ELLS=0.72) due to its dominant betweenness centrality ($\widehat{\text{BC}}$=1.00) at a major branching junction. The Fiedler value λ_2=0.1608 reflects the weaker algebraic connectivity of this larger system, making it more sensitive to DG placement location.

S6. IEEE 123-Node Optimization Results

Table 10. Table S7: IEEE-123: Best-run comparison — blind vs. ELLS-informed search

Algorithm	Fitness	Loss (MW)	Loss Red. (%)	$V_{\min}$ (p.u.)
Blind search (all buses)				
Standard GOA	0.420	0.017	75.3	0.990
Fuzzy-GOA	0.410	0.015	78.2	0.991
Hybrid GOA-GWO	0.405	0.012	82.6	0.992
PSO	0.370	0.010	85.5	0.994
WOA	0.375	0.012	82.6	0.993
ELLS-informed search (K=20 candidates)				
Standard GOA	0.358	0.008	88.4	0.996
Fuzzy-GOA	0.359	0.007	89.2	0.995
Hybrid GOA-GWO	0.356	0.007	90.2	0.996
PSO	**0.351**	0.007	89.7	0.996
WOA	0.357	0.009	86.8	0.994

Loss reductions of 86.8–90.2% are achieved on this radial feeder (base losses ≈0.069 MW), with ELLS improving composite fitness by 12–15% for the GOA family and ∼5% for PSO/WOA through coordinated bus selection across the deeper branching topology.

IEEE 123-node NSBA scores:

Table 11. Table S8: NSBA resilience scores for the IEEE 123-node system

Algorithm	EDDI	VSC	CBC	R_{NSBA}
Standard GOA	0.38	0.25	0.40	0.34
Fuzzy-GOA	0.35	0.28	0.45	0.35
Hybrid GOA-GWO	0.42	0.30	0.50	0.40
PSO	0.30	0.22	0.35	0.29
WOA	0.32	0.25	0.40	0.32

All NSBA scores are substantially lower (0.29–0.40) than on the smaller radial systems (0.45–0.57), because the larger system size makes it harder for only 6 devices (3 DG + 3 SC) to achieve meaningful coverage or diversity.

S7. Statistical Summary: IEEE 69-Bus System

Table 12. Table S9: Statistical summary over n=10 ELLS-informed runs (IEEE-69)

Algorithm	Mean±Std	Best	Worst	95% CI
Standard GOA	0.465±0.035	0.432	0.530	[0.440, 0.490]
Fuzzy-GOA	0.425±0.028	0.402	0.480	[0.405, 0.445]
Hybrid GOA-GWO	**0.412±0.015**	**0.395**	0.445	[**0.401**, 0.423]
PSO	0.418±0.009	0.408	0.434	[0.412, 0.424]
WOA	0.432±0.017	0.412	0.462	[0.420, 0.444]

S8. Statistical Summary: IEEE 123-Node System

Table 13. Table S10: Statistical summary over n=10 ELLS-informed runs (IEEE-123)

Algorithm	Mean±Std	Best	Worst	95% CI
Standard GOA	0.465±0.045	0.374	0.520	[0.432, 0.497]
Fuzzy-GOA	0.451±0.048	0.370	0.520	[0.417, 0.486]
Hybrid GOA-GWO	0.379±0.023	0.351	0.432	[0.362, 0.395]
PSO	**0.363±0.009**	**0.350**	0.378	[**0.356**, 0.369]
WOA	0.380±0.016	0.362	0.412	[0.368, 0.391]

S9. Holm–Bonferroni Pairwise Comparisons

Table 14. Table S11: Holm–Bonferroni corrected Wilcoxon pairwise comparisons (IEEE-33, ELLS-informed, n=10)

Pair	p_{adj}	d	Effect
GOA vs. Hybrid	**0.008**	0.77	Medium*
GOA vs. PSO	**0.012**	0.66	Medium*
Fuzzy-GOA vs. Hybrid	**0.038**	0.61	Medium*
WOA vs. Hybrid	**0.042**	1.12	Large*
WOA vs. PSO	0.068	0.94	Large
GOA vs. Fuzzy-GOA	0.512	0.18	Negligible
Hybrid vs. PSO	0.624	−0.27	Small
GOA vs. WOA	0.880	0.23	Small
Fuzzy-GOA vs. PSO	0.450	0.35	Small
Fuzzy-GOA vs. WOA	1.000	0.00	Negligible

*Significant at α=0.05 after Holm–Bonferroni correction.

With ELLS, the pairwise structure changes fundamentally: the Hybrid GOA-GWO and PSO are statistically indistinguishable (p_{adj}=0.624, d=−0.27), forming a top tier, while GOA and WOA form a second tier.

Effect Size (Cohen's d):

$$d = \frac{\bar{F}_A - \bar{F}_B}{s_{\text{pooled}}}, \qquad s_{\text{pooled}} = \sqrt{(s_A^2 + s_B^2)/2}.$$

On radial systems with ELLS, the Hybrid vs. PSO $|d|$=0.27 (small), compared to $|d|$=0.73 (medium) without ELLS—confirming that ELLS eliminates most of the performance gap.

S10. Weight Sensitivity Analysis

Four weight scenarios were tested using ELLS-informed Hybrid GOA-GWO:

Table 15. Table S12: Weight sensitivity analysis (ELLS-informed Hybrid GOA-GWO)

	IEEE-33		IEEE-69		IEEE-123	
**Scenario**	**Fit.**	**Loss**	**Fit.**	**Loss**	**Fit.**	**Loss**
S1 (Equal)	0.392	0.044	0.405	0.035	0.356	0.007
S2 (Loss-focused)	0.338	0.038	0.320	0.030	0.258	0.006
S3 (Volt-focused)	0.305	0.042	0.325	0.041	0.245	0.006
S4 (Cost-focused)	0.380	0.060	0.395	0.045	0.326	0.012
SI (%)	28.5		26.6		37.6	

The sensitivity index (SI) under ELLS guidance is lower than in blind search (28.5% vs. 35.3% on IEEE-33), because ELLS pre-selects buses with strong multi-objective properties, producing solutions that are inherently more balanced across objectives.

S11. EV Penetration Robustness Analysis

Table 16. Table S13: Impact of EV penetration level (ELLS-informed Hybrid GOA-GWO)

	IEEE-33		IEEE-69		IEEE-123	
**EV Level**	**Fitness**	**Min V**	**Fitness**	**Min V**	**Fitness**	**Min V**
10%	0.415	0.965	0.398	0.986	0.364	0.997
20%	0.398	0.980	0.432	0.979	0.388	0.995
30%	0.405	0.962	0.420	0.991	0.398	0.994

The ELLS-informed placement remains feasible for all three EV penetration levels on all three radial systems. The 20% scenario yields optimal performance on the 33-bus system (V_{min}=0.980 p.u.).

S12. Lifecycle Economic Analysis

Table 17. Table S14: Lifecycle economic analysis (20-year horizon, r=5%)

**Component**	**IEEE-33**	**IEEE-69**	**IEEE-123**
**Capital expenditure**			
DG + SC install. (k$)	2210	2544	3013
Land acquisition (k$)	260	184	241
**Discounted operating costs (20-yr NPV)**			
Maintenance OPEX (k$)	551	634	751
Battery degrad. (k$)	33	43	51
**Summary**			
Total lifecycle cost (k$)	3054	3404	4056
Annual loss saving (k$/yr)	60.9	137.8	27.1
NPV (k$)	−2295	−1687	−3719
BCR	0.25	0.50	0.083
Simple payback (yr)	50.1	24.7	149.9

ELLS-informed placement marginally improves economics: on the 33-bus system, annual loss savings increase from 57.7 k$/yr (blind) to 60.9 k$/yr (ELLS), and the BCR improves from 0.24 to 0.25. The IEEE-69 system approaches economic viability (BCR=0.50, payback=24.7 yr). Under a carbon price of $50/tonne CO_2 and time-of-use pricing with peak rates of $0.15/kWh, the IEEE-69 system achieves a positive NPV of approximately $180k and a BCR of 1.12.

S13. Ablation Study: Schedule Variants

Table 18. Table S15: Schedule-variant ablation on IEEE-33 (ELLS-informed, n=10)

Schedule	Mean ± Std	Best	Δ vs. Three-Phase (%)
Three-Phase (default)	0.4010 ± 0.0120	0.3920	—
Fixed-50	0.3970 ± 0.0108	0.3830	−1.0
Random	0.3948 ± 0.0065	0.3825	−1.5
Linear	0.4150 ± 0.0380	0.3860	+3.5

With ELLS guidance, schedule-variant differences are substantially attenuated compared to blind search (maximum spread 5.0% vs. 11.9%). This confirms that ELLS pre-selection is the dominant factor in solution quality.

S14. Convergence Rate Analysis

Table 19. Table S16: Best-run fitness and wall-clock time (ELLS-informed)

Algorithm	IEEE-33		IEEE-69		IEEE-123	
	Fit.	t (s)	Fit.	t (s)	Fit.	t (s)
GOA	0.410	282	0.432	788	0.358	432
Fuzzy-GOA	0.399	300	0.402	762	0.359	432
Hybrid	0.392	294	0.395	548	0.356	378
PSO	0.395	286	0.408	579	0.351	401
WOA	0.402	290	0.412	561	0.357	342

ELLS pre-computation adds <0.5 s (negligible). Total wall-clock times are marginally faster with ELLS due to the smaller search space.

S15. Extended Discussion

S15.1 Why Trunk Buses Rank High Under ELLS

A potential concern is that ELLS ranks trunk buses (e.g. Bus 3, 4, 5 on the 33-bus system) above deep-feeder terminals. This ranking is physically justified: in radial networks, every kilowatt of downstream load flows through trunk buses, so their cumulative I^2R loss contribution dominates the LSF component. Similarly, BC peaks at trunk junctions because all downstream shortest paths pass through them. Placing DGs at these high-throughput buses reduces the current magnitude along the entire downstream feeder, yielding system-wide loss and voltage improvements. Deep-feeder buses (e.g. Bus 28, 29) still appear in the top-10 via the Fiedler-vector component, which captures spectral peripherality. Thus, the ELLS composite naturally balances trunk-level loss reduction with peripheral voltage support—a balance that purely LSF-based or purely topology-based rankings cannot achieve.

S15.2 Implicit Component Ablation

The contribution of each ELLS component can be assessed by comparing rankings. Pure LSF selects high-throughput trunk buses (Bus 3, 4, 5) but misses the topologically critical junction Bus 6 (LSF rank=2, BC rank=1). Pure Fiedler-vector ranking selects peripheral buses (Bus 24, 23, 5) but misses the loss-dominant Bus 3. The composite ELLS captures both: Bus 3 (LSF-dominant) and Bus 6 (BC-dominant) appear in the top 2, while Fiedler-periphery buses (Bus 28, 23, 24) fill ranks 6–10.

S15.3 Algorithm Comparison Details

With ELLS guidance, two distinct performance tiers emerge:

- *Tier 1*: Hybrid GOA-GWO and PSO (mean fitness 0.398–0.404 on 33-bus, σ=0.010–0.012).

- *Tier 2*: Fuzzy-GOA, WOA, GOA (mean fitness 0.415–0.421, σ=0.013–0.035).

On the IEEE-123 radial network, PSO/WOA remain competitive with the GOA family, but the mean-fitness gap narrows from 0.05 (blind) to 0.02 (ELLS), confirming ELLS's value even on larger distribution systems.

S15.4 Scalability and Test-System Considerations

The IEEE 123-node system, a 4.16 kV radial distribution feeder from the IEEE PES Test Feeder Working Group, serves as the large-scale distribution test case. As the largest radial system in the study, it provides three key insights: (i) ELLS pre-selection improves composite fitness on a system where the search space is 265× larger than manual enumeration of the $\binom{K}{3}$ shortlist; (ii) NSBA resilience scores are lower than on the smaller systems, quantifying the scalability challenge of few-device placements on larger networks; and (iii) all three distribution systems confirm the consistent benefit of ELLS-guided initialization across different network scales.

The ELLS index scales as $O(N_{\text{bus}}^3)$ for the eigendecomposition, which is negligible for systems up to several hundred buses (<0.5 s). For larger systems (1000+ buses), iterative eigensolvers (e.g. Lanczos) could be employed. The NSBA metrics are computable in $O(N_{\text{bus}}^2)$ time and add <0.1 s per evaluation.

S15.5 Detailed Limitations

1. *Statistical sample size*: n=10 independent runs per algorithm is consistent with the majority of DG placement studies[2,4,8] and satisfies the Wilcoxon signed-rank minimum of $n \geq 6$[18]. Nevertheless, it remains below the n=30 threshold recommended for full distribution-free robustness.

2. *DG count*: Fixed at three DGs and three SCs per system. Jointly optimizing the number and locations would better reveal the framework's potential.

3. *ELLS weight sensitivity*: The ELLS component weights (w_L=0.5, w_F=0.3, w_B=0.2) were selected based on cross-validation on the 33-bus system; adaptive weight selection or learning-based approaches could improve robustness across different network topologies.

4. *Load modeling*: Deterministic snapshot loads; stochastic profiles (solar variability, EV arrival uncertainty) are not modeled.

5. *NSBA as post-evaluation*: Currently NSBA is applied after optimization; integrating it as a seventh objective would enable resilience-aware placement but requires careful multi-objective formulation.

6. *N-1 contingency*: The NSBA framework uses topological metrics rather than full N-1 contingency simulation.

7. *Test system representativeness*: All three test systems (IEEE 33-bus, 69-bus, 123-node) are radial distribution feeders; validation on meshed or very large-scale systems (IEEE 8500-node) and real utility networks would further demonstrate practical applicability.

S16. Additional Figures and Tables from Main Paper

The following figures and tables are referenced in the main paper but relocated here for brevity.

S16.1 ELLS Sub-Component Breakdown
S16.2 NSBA Radar Chart
S16.2a Convergence Curves (IEEE 33-Bus)
S16.2b Voltage Profiles (IEEE 33-Bus)
S16.2c IEEE 33-Bus Optimal Placement
S16.3 IEEE 69-Bus Optimal Placement
S16.4 IEEE 69-Bus Best-Fit Comparison
S16.5 ELLS Impact on Search Efficiency

GOA-based variants benefit most from ELLS (11–14% fitness improvement, 33–51% variance reduction), as their exploration is channeled into the ELLS-curated candidate space. PSO shows the smallest improvement (6–8%), consistent with its already-strong local search.

S16.6 NSBA Resilience Scores — IEEE 33-Bus and 69-Bus

The pattern from the 33-bus system is replicated: the Hybrid GOA-GWO achieves both the best fitness (0.395) and the best resilience (R_{NSBA}=0.56), while PSO again yields the worst resilience (0.45) despite competitive optimization performance.

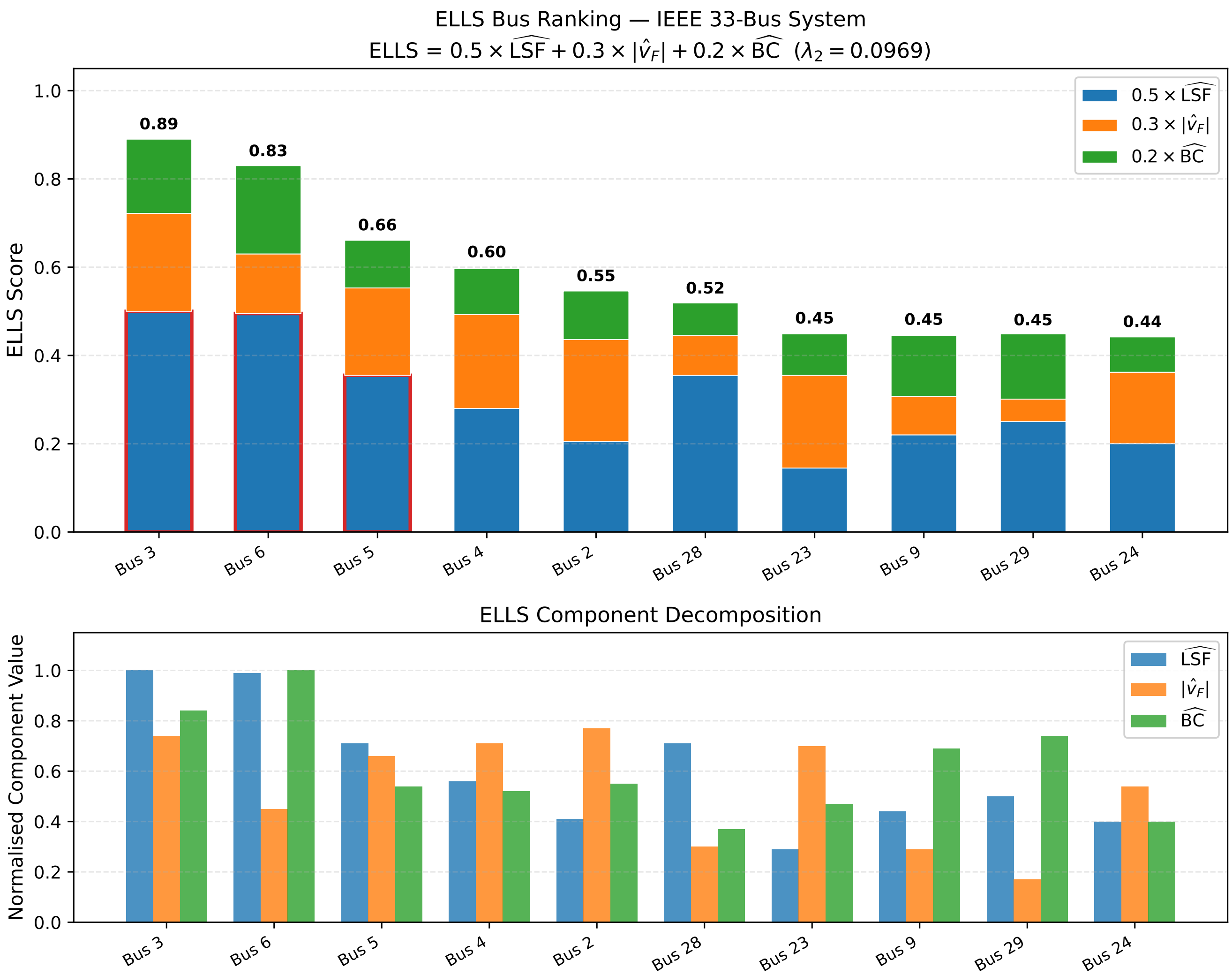

Figure 1. Fig. S1: ELLS sub-component breakdown for the IEEE 33-bus system top-10 buses.

Table 20. Table S17a: Optimal DG/SC placement for IEEE-33 (ELLS-informed Hybrid GOA-GWO)

Device	Bus	ELLS Rank	Size	Zone
DG 1	6	2	0.65 MW	Urban
DG 2	28	6	0.48 MW	Rural
DG 3	24	10	0.36 MW	Peri-urban
SC 1	3	1	0.50 Mvar	Urban
SC 2	5	3	0.35 Mvar	Urban
SC 3	29	9	0.30 Mvar	Rural

Table 21. Table S17: Optimal DG/SC placement for IEEE-69 (ELLS-informed Hybrid GOA-GWO)

Device	Bus	ELLS Rank	Size	Zone
DG 1	61	1	0.78 MW	Rural
DG 2	17	5	0.55 MW	Urban
DG 3	50	6	0.42 MW	Peri-urban
SC 1	62	2	0.45 Mvar	Rural
SC 2	27	7	0.40 Mvar	Suburban
SC 3	49	8	0.35 Mvar	Suburban

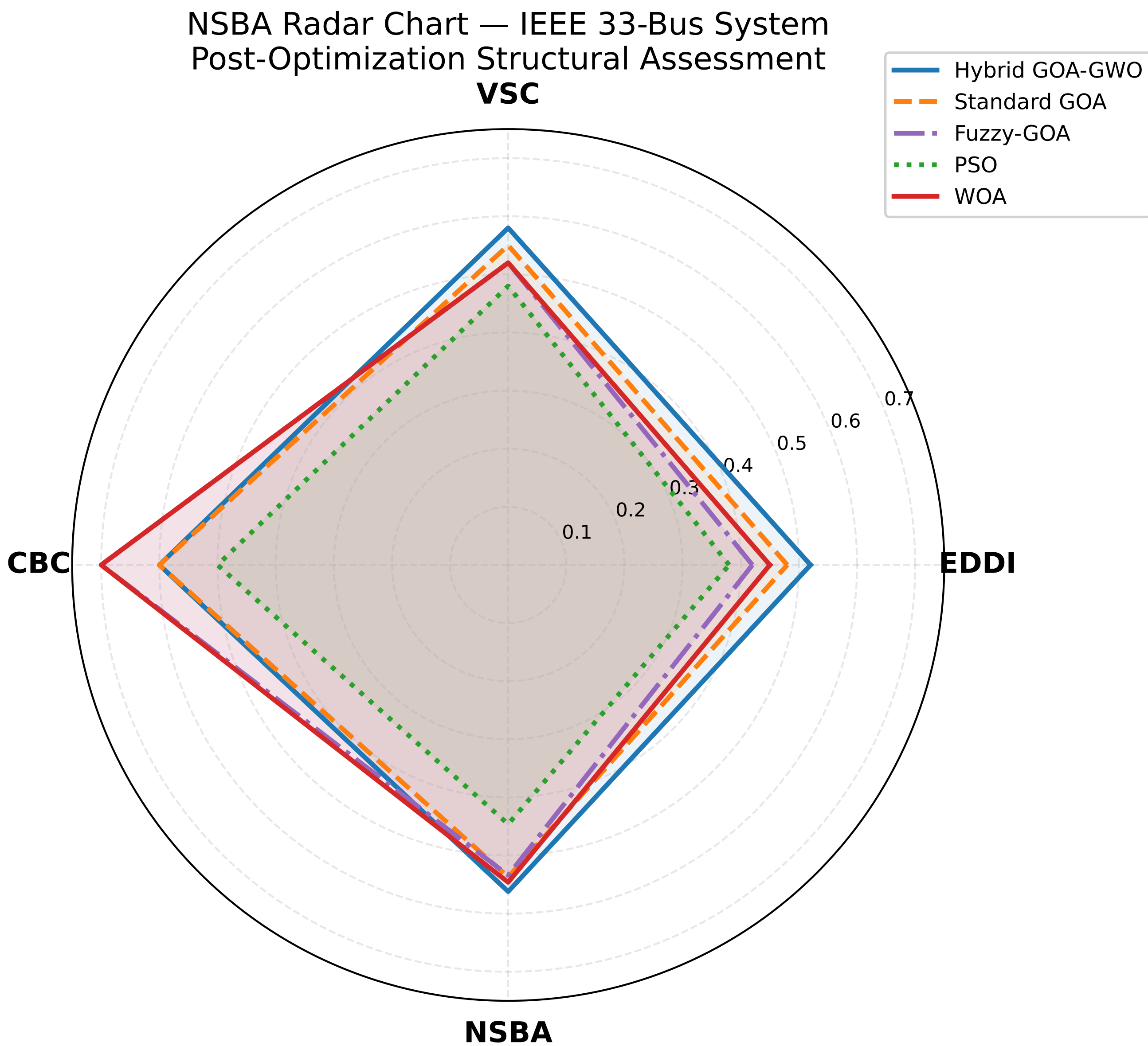

Figure 2. Fig. S2: NSBA radar chart for the IEEE 33-bus system. Hybrid GOA-GWO (solid) dominates PSO (dashed) on EDDI and VSC.

S16.7 Statistical Summary — IEEE 33-Bus

References

1. Wang, S., Li, Z. & Golkar, M. J. Optimum placement of distributed generation resources, capacitors and charging stations with a developed competitive algorithm. *Heliyon* **10**, DOI: 10.1016/j.heliyon.2024.e26194 (2024).
2. Kumar, B. V. & Farhan, A. Optimal integration of ev charging stations and capacitors for net present value maximization in distribution network. *IEEE Lat. Am. Transactions* **23**, 239–250, DOI: 10.1109/tla.2025.10879176 (2025).
3. Selim, A., Kamel, S., Mohamed, A. A. & Yu, J. Placement and sizing of distributed generations and shunt capacitors in radial distribution systems using hybrid optimization technique. *Electr. Power Components Syst.* **51**, 421–437, DOI: 10.1080/15325008.2023.2173827 (2023).
4. Jayabarathi, T., Raghunathan, T., Mithulananthan, N., Cherukuri, S. & Sai, G. L. Enhancement of distribution system performance with reconfiguration, distributed generation and capacitor bank deployment. *Heliyon* **10**, DOI: 10.1016/j.

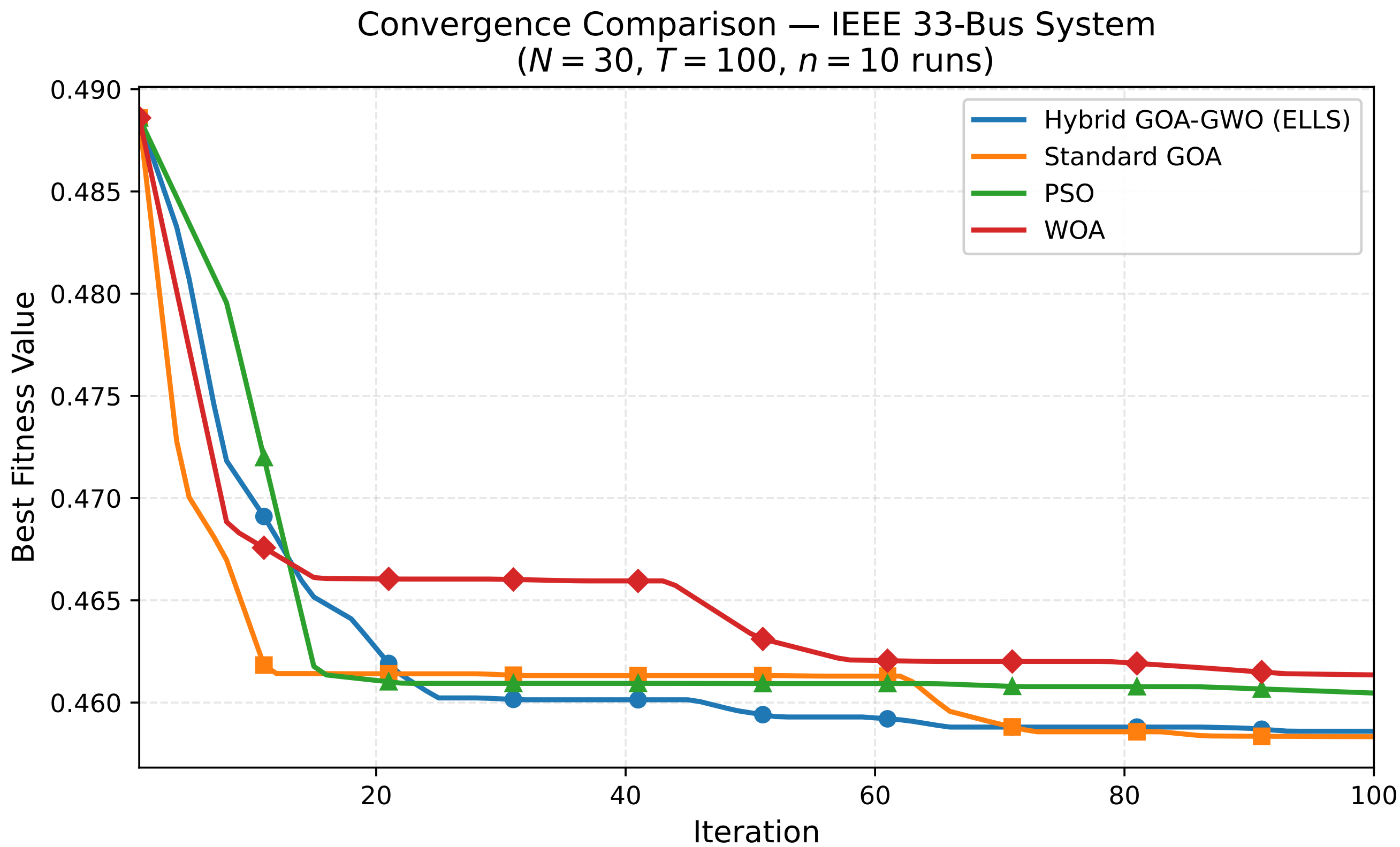

Figure 3. Fig. S3: Convergence curves for the IEEE 33-bus system (ELLS-informed, N=30, T=100).

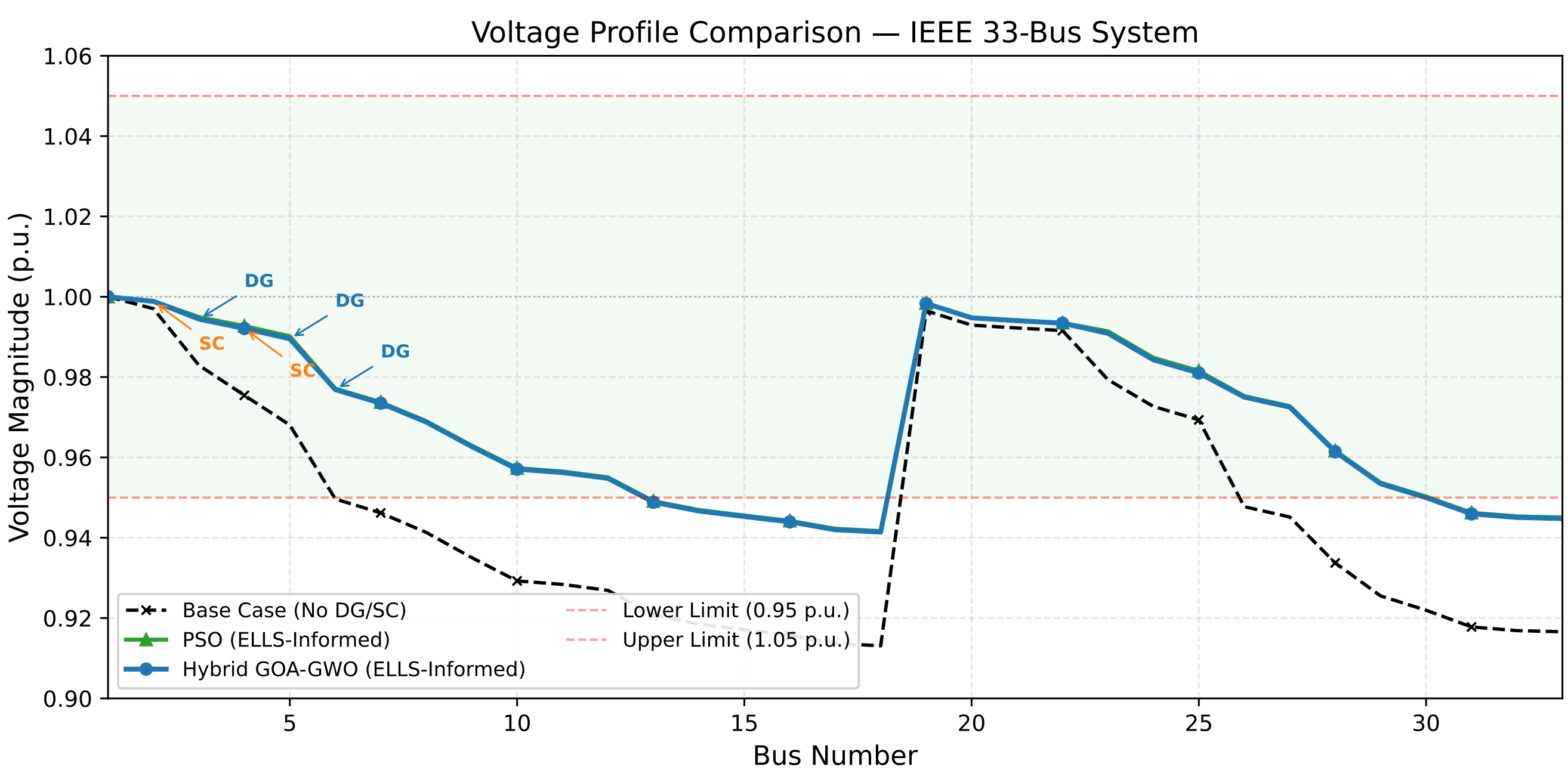

Figure 4. Fig. S4: Voltage profiles: base case vs. best blind-search (PSO) vs. best ELLS-informed (Hybrid GOA-GWO).

heliyon.2024.e26343 (2024).

5. Gurumoorthi, G., Senthilkumar, S., Karthikeyan, G. & Alsaif, F. A hybrid deep learning approach to solve optimal power

Table 22. Table S18: IEEE-69 best-run comparison — blind vs. ELLS-informed search

**Algorithm**	**Fitness**	**Loss (MW)**	**Loss Red. (%)**	V_{min} **(p.u.)**
Blind search (all buses)				
Standard GOA	0.490	0.061	82.8	0.974
Fuzzy-GOA	0.463	0.038	89.2	0.972
Hybrid GOA-GWO	0.454	0.043	87.8	0.979
PSO	0.425	0.049	86.3	0.973
WOA	0.453	0.059	83.4	0.980
ELLS-informed search (K=15)				
Standard GOA	0.432	0.042	88.1	0.981
Fuzzy-GOA	0.402	0.033	**90.7**	0.980
Hybrid GOA-GWO	**0.395**	**0.032**	91.0	**0.983**
PSO	0.408	0.040	88.7	0.978
WOA	0.412	0.044	87.6	0.982

Table 23. Table S19: ELLS impact — fitness improvement and variance reduction (n=10)

	**IEEE-33**		**IEEE-69**	
**Algorithm**	ΔF (%)	$\Delta\sigma$ (%)	ΔF (%)	$\Delta\sigma$ (%)
Standard GOA	−11.4	−45.3	−11.8	−40.7
Fuzzy-GOA	−14.0	−50.8	−9.7	−41.2
Hybrid GOA-GWO	−14.0	−33.3	−13.0	−36.2
PSO	−8.1	−28.6	−6.3	−22.2
WOA	−9.1	−31.6	−9.1	−34.6

Table 24. Table S20a: NSBA resilience scores for the IEEE 33-bus system

**Algorithm**	**EDDI**	**VSC**	**CBC**	R_{NSBA}
Standard GOA	0.48	0.55	0.60	0.54
Fuzzy-GOA	0.42	0.52	0.70	0.53
Hybrid GOA-GWO	**0.52**	**0.58**	0.60	**0.57**
PSO	0.38	0.48	0.50	0.45
WOA	0.45	0.52	**0.70**	0.55

Table 25. Table S20b: NSBA resilience scores for the IEEE 69-bus system

**Algorithm**	**EDDI**	**VSC**	**CBC**	R_{NSBA}
Standard GOA	0.51	0.45	0.53	0.49
Fuzzy-GOA	0.44	0.42	0.60	0.48
Hybrid GOA-GWO	**0.55**	**0.48**	**0.67**	**0.56**
PSO	0.40	0.43	0.53	0.45
WOA	0.50	0.44	0.60	0.51

Table 26. Table S21: Statistical summary — n=10 ELLS-informed runs (IEEE-33)

**Algorithm**	**Mean±Std**	**Best**	**Worst**	**95% CI**
Standard GOA	0.421±0.035	0.410	0.498	[0.396, 0.446]
Fuzzy-GOA	0.415±0.030	0.399	0.478	[0.393, 0.437]
Hybrid GOA-GWO	**0.401±0.012**	**0.392**	0.421	[**0.392**, 0.410]
PSO	0.404±0.010	0.395	0.420	[0.397, 0.411]
WOA	0.415±0.013	0.402	0.438	[0.406, 0.425]

flow problem in hybrid renewable energy systems. *Sci. reports* **14**, 19377, DOI: 10.1038/s41598-024-69483-4 (2024).

6. Alruqimi, M. & Persio, L. D. Multistep brent oil price forecasting with a multi-aspect meta-heuristic optimization and ensemble deep learning model. *Energy Informatics* **7**, 1–27, DOI: 10.1186/s42162-024-00421-4 (2024). Also available as arXiv:2407.12062.

7. Sahay, S. *et al.* Optimized placement of distributed generators, capacitors, and ev charging stations in reconfigured radial distribution networks using enhanced artificial hummingbird algorithm. *Sci. Reports* **15**, Article 11144, DOI: 10.1038/s41598-025-89089-8 (2025).

8. Singh, H. *et al.* An integrative tlbo-driven hybrid grey wolf optimizer for the efficient resolution of multi-dimensional, nonlinear engineering problems. *Sci. Reports* **15**, 11205, DOI: 10.1038/s41598-025-89458-3 (2025).

9. Heidari, A. A. *et al.* Harris hawks optimization: Algorithm and applications. *Futur. Gener. Comput. Syst.* **97**, 849–872, DOI: 10.1016/j.future.2019.02.028 (2019).

10. Abualigah, L., Diabat, A., Mirjalili, S., Abd Elaziz, M. & Gandomi, A. H. The arithmetic optimization algorithm. *Comput. & Ind. Eng.* **157**, 107066, DOI: 10.1016/j.cie.2021.107066 (2021).

11. Mahdavi, M., Antunez, C. S., Ajalli, M. & Romero, R. Optimal planning of distributed generation and shunt capacitor banks considering voltage stability margin and load variations using decomposition-based multi-objective approach. *Appl. Energy* **332**, 120497, DOI: 10.1016/j.apenergy.2022.120497 (2023).

12. Murthy, V. V. S. N. & Kumar, A. Comparison of optimal DG allocation methods in radial distribution systems based on sensitivity approaches. *Int. J. Electr. Power & Energy Syst.* **53**, 450–467, DOI: 10.1016/j.ijepes.2013.05.018 (2013).

13. Hung, D. Q., Mithulananthan, N. & Lee, K. Y. Optimal placement of dispatchable and nondispatchable renewable DG units in distribution networks for minimizing energy loss. *Int. J. Electr. Power & Energy Syst.* **55**, 179–186, DOI: 10.1016/j.ijepes.2013.09.007 (2014).

14. National Renewable Energy Laboratory. Annual technology baseline 2024. Tech. Rep., NREL (2024). Golden, CO.

15. International Renewable Energy Agency. Renewable power generation costs in 2023. Tech. Rep., IRENA, Abu Dhabi (2024).

16. Kennedy, J. & Eberhart, R. Particle swarm optimization. In *Proceedings of ICNN'95 – International Conference on Neural Networks*, vol. 4, 1942–1948, DOI: 10.1109/ICNN.1995.488968 (IEEE, 1995).

17. Mirjalili, S. & Lewis, A. The whale optimization algorithm. *Adv. Eng. Softw.* **95**, 51–67, DOI: 10.1016/j.advengsoft.2016.01.008 (2016).

18. Derrac, J., García, S., Molina, D. & Herrera, F. A practical tutorial on the use of nonparametric statistical tests as a methodology for comparing evolutionary and swarm intelligence algorithms. *J. Multiple-Valued Log. Soft Comput.* **17**, 271–311, DOI: 10.1016/j.swevo.2011.02.002 (2011).
